# Immobilizing Zwitterionic Molecular Brush in Functional Organic Interfacial Layers for Ultra-Stable Zn-Ion Batteries

**DOI:** 10.1007/s40820-025-01782-5

**Published:** 2025-05-20

**Authors:** Limeng Sun, Xianjun Cao, Li Gao, Jiayi Li, Chen Qian, Jinhu Wu, Xinming Nie, Hong Gao, Peng Huang, Yufei Zhao, Yong Wang, Jinqiang Zhang, Guoxiu Wang, Hao Liu

**Affiliations:** 1 Joint International Laboratory on Environmental and Energy Frontier Materials, School of Environmental and Chemical Engineering, Shanghai University, Shanghai, 200444 People’s Republic of China; 2 Centre for Clean Energy Technology, Faculty of Science, University of Technology Sydney, Broadway, Sydney, NSW 2007 Australia; 3 School of Physics and Electronic Engineering, Jiangsu Normal University, Xuzhou, 221116 Jiangsu People’s Republic of China; 4 School of Chemistry and Materials Science, Jiangsu Normal University, Xuzhou, 221116 Jiangsu People’s Republic of China

**Keywords:** Zinc-ion batteries, Zn anodes, Functional organic interfacial layers, Electrolyte design, Organic-assist SEI pre-construction

## Abstract

Rechargeable zinc-ion batteries have emerged as one of the most promising candidates for large-scale energy storage applications due to their high safety and low cost. However, the use of Zn metal in batteries suffers from many severe issues, including dendrite growth and parasitic reactions, which often lead to short cycle lives. Herein, we propose the construction of functional organic interfacial layers (OIL) on the Zn metal anodes to address these challenges. Through a well-designed organic-assist pre-construction process, a densely packed artificial layer featuring the immobilized zwitterionic molecular brush can be constructed, which can not only efficiently facilitate the smooth Zn plating and stripping, but also introduce a stable environment for battery reactions. Through density functional theory calculations and experimental characterizations, we verify that the immobilized organic propane sulfonate on Zn anodes can significantly lower the energy barrier and increase the kinetics of Zn^2+^ transport. Thus, the Zn metal anode with the functional OIL can significantly improve the cycle life of the symmetric cell to over 3500 h stable operation. When paired with the H_2_V_3_O_8_ cathode, the aqueous Zn-ion full cells can be continuously cycled over 7000 cycles, marking an important milestone for Zn anode development for potential industrial applications.

**Supplementary Information:**

The online version contains supplementary material available at 10.1007/s40820-025-01782-5.

## Highlights

- An organic-assisted solid electrolyte interface pre-construction creates a functional organic interfacial layer (OIL-IPS@Zn) with aligned zwitterionic structures, suppressing side reactions and enhancing Zn^2+^ transport.
- OIL-IPS@Zn shows superior electrolyte affinity, enabling symmetric cells to operate stably for 3500 h at 1 mA cm^−2^/1 mAh cm^−2^ and 3200 h at 50 mA cm^−2^/10 mAh cm^−2^.
- With 99.90% Zn plating/stripping efficiency, full cells paired with H_2_V_3_O_8_ cathodes achieve over 7000 cycles.

**Supplementary Information:**

The online version contains supplementary material available at 10.1007/s40820-025-01782-5.

## Graphical abstract

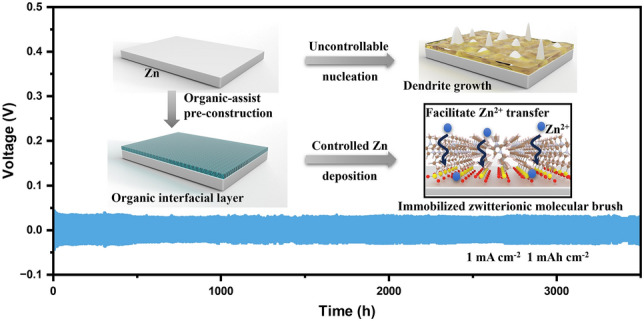

## Introduction

Electrochemical energy storage devices play a crucial role in the utilization of renewable energy sources, thereby making the development of renewable clean energy strategically important for establishing a sustainable society [1–6]. Aqueous zinc-ion (Zn-ion) batteries, which directly use metallic zinc as the anode, are considered one of the ideal batteries due to their abundance, non-toxicity, high safety, and high theoretical capacity (820 mAh g^−1^) [7–11]. Despite the extensive research on aqueous zinc-ion batteries for large-scale energy storage systems, the potential commercial application of these batteries faces various challenges, such as dendrite growth and parasitic reactions at the zinc anode, which often lead to low capacity, poor cycling performance, and even safety issues [12–17]. Therefore, it is highly desirable to develop stable Zn anodes with high plating/stripping efficiency, dendritic growth suppression, and alleviated parasitic reactions, to enhance the overall electrochemical performance and cycling stability.

Up to now, several strategies have been proposed to improve the reversibility and stability of Zn electrodes for Zn-ion batteries, including hierarchical structural design [18, 19], artificial protective layer design [20], electrolyte modification [21], and separator modification [22, 23]. In particular, introducing an artificial protective layer on the Zn anodes, such as TiO_2_ [24], BTO@Zn [25], Zn_3_(PO_4_)_2_·4H_2_O [26], and NaC(CN)_3_ [27], shows robust potential, which can significantly improve the stability of the Zn metal anode by reducing parasitic reactions and dendrite formation. For instance, Li et al*.* [28] reported the design of the MOF-CeO_2_ artificial protection layer on the zinc anode, which promoted the dissolution process of [Zn(H_2_O)_6_]^2+^ and balanced the flux of Zn ions through the pore channels, leading to improved Coulombic efficiency and cycle stability. Nevertheless, introducing foreign layers on Zn surface often faces interfacial issues that may cause insufficient contact and a low Zn^2+^ transport rate [29–31]. Therefore, in situ constructing artificial solid electrolyte interface (SEI) derived from electrolytes can be an alternative strategy to maintain excellent interfacial contact [32, 33]. However, due to the constant hydrogen evolution and the repetitive large volume changes of Zn metal, these SEI layers from aqueous electrolytes are usually not densely packed and tend to crack and detach from the Zn surface during electrochemical cycling [34]. Additionally, the SEI derived from aqueous electrolytes is usually dominated by inorganic components with low electronic/ionic conductivity, leading to low Zn^2+^ migration number and Coulombic efficiency (CE) [35]. One strategy to increase the properties of the SEI layers is to increase the organic salt concentration, which facilitates the decomposition of the organic anions to dominate the SEI with organic components. The introduction of high-concentration salts, however, may increase the viscosity of the electrolytes, leading to sluggish kinetics and low overall efficiency. On the other hand, SEI layers constructed in organic electrolytes are denser and more robust compared to the ones from aqueous electrolytes, resulting in high resistance to cracking and parasitic reactions [32, 36, 37]. Nevertheless, the direct use of organic electrolytes instead of aqueous ones for battery cycling will significantly increase the overall cost and decrease the safety of the batteries. In addition, the resistance of the SEI in organic electrolytes is still high, which may also lead to the block of Zn^2+^ transport and low electrochemical performance [36, 38]. It is widely recognized that the most efficient strategy is to utilize the organic electrolytes to facilitate the stable SEI formation [33], while operating the cell with the processed zinc anodes in the aqueous electrolytes, which can significantly overcome the disadvantages of both organic electrolytes and aqueous electrolytes [39].

Herein, we designed an organic-assist pre-construction (OAPC) process to synthesize the functional organic interfacial layers (OIL) on the Zn metal anode to increase the electrochemical performance. Through carefully controlling the conditions in modification with 1-propylsulfonate-2,3-dimethylimidazole (IPS), we construct an immobilized zwitterionic molecular brush layer within the densely packed OIL (OIL-IPS@Zn). Through density functional theory (DFT) calculations, we identified that the grafting of the zwitterionic molecular brush can significantly reduce the energy barrier for Zn^2+^ transport and plating/stripping. Combining with the capability of densely packed organic-dominated OIL to suppress parasitic reactions and dendrite growth, the unique structure of OIL-IPS@Zn can efficiently increase the affinity toward aqueous electrolytes and enhance the Zn^2+^ transport through the layers, leading to smooth Zn plating and stripping with excellent stability [40]. As a result, significant improvements in cycle stability are achieved for OIL-IPS@Zn symmetrical cells at both low and high current densities. When applied in full aqueous Zn-ion batteries, the OIL-IPS@Zn||H_2_V_3_O_8_ full cell maintains excellent capacity retention after over 7000 cycles.

## Experimental Section

### Materials

Zinc foil (99.999%, 0.1 mm) and titanium foil (99.999%, 0.02 mm) were procured from the High Purity Metals Research Institute. Acetylene black and polyvinylidene fluoride (PVDF) were sourced from Guangdong Chuglight New Energy Technology Co., Ltd. Zinc trifluoromethanesulfonate (Zn(OTf)_2_) (98%), 1,3-propanesultone (99%), and vanadium pentoxide (99%) were purchased from Macklin. Acetonitrile (AR), diethyl ether (AR), anhydrous sodium sulfate (AR), and 30% H_2_O_2_ (GR) were acquired from China National Pharmaceutical Group Corporation. 1,2-dimethylimidazole and propylene carbonate (PC) (99%), as well as Poly (Ethylene Glycol) 2000, were sourced from Adamas-beta. N-methyl-2-pyrrolidone (NMP) was obtained from GENERAL-REAGENT. Glass microfiber filter (GF/F) was procured from Whatman. Deionized water was used for preparing aqueous electrolytes. The high-purity N_2_ (Shanghai Yujiali Special Gas Co., Ltd. 99.999%) was used directly.

### Material Synthesis

#### Preparation of 1-*propylsulfonate*-2,3-*dimethylimidazole*

IPS was prepared through a ring-opening-assisted grafting method. Typically, 7 mmol (0.6729 g) 1,2-dimethylimidazole and 5 mmol (0.6107 g) 1,3-propanesultone were dissolved in 15 mL of acetonitrile. The mixture was then heated at 75 °C in a thermostatic bath for 24 h, resulting in white crystalline solids. Upon cooling, both the crystals and the supernatant were transferred into diethyl ether and washed three times. The obtained solid was then placed in a vacuum and dried oven at room temperature to obtain a white powder.

#### Preparation of the OIL-IPS@Zn, OIL@Zn and ADL@Zn

The OIL was prepared through the designed OAPC process by assembling the symmetric cells with Zn anodes and organic electrolytes. The electrolytes were prepared by mixing propylene carbonate and water in certain ratios, along with the addition of 0.5 M Zn(OTf)_2_. The symmetric cells were discharged/charged at the current density of 1 mA cm^−2^ and the capacity density of 1 mAh cm^−2^ for certain cycles to pre-treat the Zn electrodes. The obtained Zn electrodes were taken out from the cells and cleaned with pure water 3 times to remove any residue of the organic electrolytes before dried under vacuum. The OIL-IPS@Zn was prepared through this process with the addition of IPS, while the OIL@Zn was prepared without IPS. The ADL@Zn was prepared through a similar process with aqueous electrolyte (0.5 M Zn(OTf)_2_ in water) instead of organic electrolytes.

#### Preparation of Cathode Materials for Full Cell Tests

The H_2_V_3_O_8_ powder was synthesized via a hydrothermal method. Typically, the synthesis procedure involved dissolving 1.0 g of V_2_O_5_ in 40 mL of deionized water, followed by stirring the mixture at room temperature for 2 h. Subsequently, 12 mL of H_2_O_2_ was added, and the mixture was stirred for 3 h. The solution is then transferred to a 100 mL reaction vessel and heated at 180 °C for 2 days. The resulting product was washed three times with deionized water and ethanol, and the obtained powder was dried in a vacuum oven at 80 °C for 12 h until complete evaporation of water. The obtained powder was calcined at 250 °C in a tube furnace under a nitrogen atmosphere with a heating rate of 5 °C min^−1^ for 3 h. Subsequently, the sample was cooled to room temperature and ground for 30 min to obtain the H_2_V_3_O_8_ sample. The anodes were prepared by mixing H_2_V_3_O_8_ powder, acetylene black, and PVDF in a 7:2:1 mass ratio in an NMP solution and stirring for 30 min. The resulting slurry was then uniformly coated onto a titanium foil using a scraper and dried at 80 °C in a vacuum drying oven for 12 h.

### Characterizations

The electrode morphology was analyzed with Japanese JEOL JEM-7500F scanning electron microscope (SEM) at 5 kV voltage and 10 μA current, and was mapped with energy dispersive spectrometer (EDS) of OXFORD Company, UK. Before SEM examination, the cycled Zn anode underwent a cleaning process by immersion in deionized water three times followed by vacuum drying. X-ray diffraction (XRD) spectra were obtained using a Japanese Rigaku D/max-2200 instrument with a scanning range from 5 to 85°. Fourier-transform infrared (FTIR) spectra were acquired using a US Thermo Scientific Nicolet iS20 FTIR spectrometer. High-resolution mass spectrometry (HRMS) data were obtained using a US Agilent 1260-6530A instrument with a mass scan range of 50–1200 m z^−1^. Organic elemental analysis data were acquired using a US Thermo Scientific Flash 2000 Elemental analyzer (EA) in CHNS mode. UV spectra were obtained using a TU-1901 instrument with a scanning range from 190 to 700 nm. Contact angle measurements comparing the wettability of deionized water and 0.5 M Zn(OTf)_2_ electrolyte on the surfaces of bare Zn, ADL@Zn, OIL@Zn, and OIL-IPS@Zn electrodes were conducted using an OCA 25 contact angle tester through static contact angle measurements. Atomic force microscopy (AFM) images were obtained using a Korean Park Systems XE7 operating in tapping mode. X-ray photoelectron spectroscopy (XPS) was obtained by Thermo Scientific ESCALAB 250Xi X-ray photoelectron spectrometer. Using Bruker 600 M Nuclear magnetic resonance (NMR) spectrometer, the NMR hydrogen spectrum was obtained by scanning 8 times.

### Electrochemical Measurements

All batteries were assembled and disassembled under ambient conditions and tested using 2032 coin-type cells. Zn symmetric cells were used to evaluate the stability of Zn anodes, while Cu||Zn cells were employed to assess the Coulombic efficiency (CE) of Zn plating and stripping. All assembled Zn||H_2_V_3_O_8_ cells were allowed to stand for 12 h before testing. Electrochemical measurements including cyclic voltammetry (CV), electrochemical impedance spectroscopy (EIS), Tafel curves, and chronoamperometry (CA) were conducted using a CHI760E electrochemical workstation (CH Instruments, Shanghai, China). EIS test using stainless steel symmetric cells was conducted over the frequency range of 10 mHz–100 kHz, and the ionic conductivity is calculated with Eq. (1):

1 $$\sigma = \frac{1}{RS}$$

where l is the electrolyte thickness, R denotes the bulk resistance, and S denotes the area of the electrode. The transference number of Zn^2+^ (t_Zn2+_) was performed by chronoamperometry and calculated by Eq. (2):

2 $$t_{{{\text{Zn}}2 + }} = \frac{{I_{S} \left( {\Delta V - I_{0} R_{0} } \right)}}{{I_{0} \left( {\Delta V - I_{S} R_{S} } \right)}}$$

where *I*_0_ and *R*_0_ represent the current and resistance before polarization, *I*_*S*_ and *R*_*S*_ represent the current and resistance after polarization, and ΔV corresponds to the applied polarization potential (10 mV). CA and Tafel curve measurements utilized a three-electrode system with Bare Zn or OIL@Zn or OIL-IPS@Zn as the working electrode, Pt foil as the counter electrode, Ag/AgCl as the reference electrode, 1 M Na_2_SO_4_ as the electrolyte, and a voltage range of − 0.8 to − 2.0 V. CV curves for symmetric cells were obtained within the range of − 0.1 to 0.1 V, for Cu||Zn cells within the range of − 0.2 to 0.2 V, and for Zn||H_2_V_3_O_8_ cells within the range of 0.2 to 1.6 V. All cycling tests for symmetric cells, half cells, and full cells were conducted on a battery testing system (NEWARE, Shenzhen, China). Current density and specific capacity were calculated based solely on cathode mass for consistency.

### In Situ Optical Microscope

The in situ optical microscope monitoring the Zn plating on the Zn surface was recorded by biological microscope (Smartzoom 5, Carl Zeiss AG, Germany). A home-made cell was fabricated for the testing, in which two Zn chips were attached on two sides of a flume of a glass pane, and electrolytes were used in the flume. During the test, a constant current (5 mA cm^−2^, based on the area of cross-section of Zn chips) was applied for the Zn plating by using an Arbin BT2000 potentiostat (LAND Electronics, Wuhan, China).

### Attenuated Total Reflection Surface-enhanced Infrared Absorption Spectroscopy (ATR-SEIRAS)

ATR-SEIRAS was conducted using a Nicolet iS50 FTIR spectrometer equipped with a narrow band MCT-A detector and an in situ IR optical accessory (SPEC-I, Shanghai Yuanfang Tech.) at an incidence angle of ca. 60°. Semi-cylindrical zinc selenide crystals are used for specular reflection components. The interferometer and the specular reflection assembly were purged with high-purity N_2_ (99.999%) during the whole experimental process. The IR spectra were collected with unpolarized IR radiation at the spectral resolution of 8 cm^−1^. All spectra were shown in absorbance, defined as − log(*R*/*R*_0_), where R and R_0_ represent the sample and reference single-beam spectra, respectively. Galvanostatic charge–discharge was carried out using an Arbin BT2000 potentiostat (LAND Electronics, Wuhan, China).

### Calculation Methods

All DFT calculations were implemented by the Vienna Ab initio simulation package (VASP) [41]. The electron exchange and correlation energies were handled using the Perdew–Burke–Ernzerhof (PBE) functionals [42]. The projector augmented wave (PAW) potentials [43, 44] was used to describe the interactions between the cores and valence electrons. The expansion of the Kohn–Sham valence states was carried out with a 400 eV plane-wave cutoff energy. For Brillouin zone integration, 3 × 3 × 3 Γ-centered Monkhorst–Pack grids were performed. The Zn (002) surface unit cells were four layers thick, and the bottom two layers were fixed to the bulk position of Zn. The convergence criterion of structure optimization for energy and force were set as 10^−4^ eV and 0.03 eV Å^−1^, respectively. Constrained ab initio molecular dynamics (cAIMD) simulations with a SG sampling approach [45, 46] as implemented in VASP (SG-AIMD) are performed to evaluate the kinetic process of desolvation of zinc ions with imidazole. During SG-AIMD, the bottom two layers of the Zn metal substrate were fixed. Due to the complexity of the SEI composition on the zinc anodes, we simplified the models by constructing water layers with the same thickness on the zinc metal surface. For the case of OIL-IPS, we added uniformly distributed IPS in the water layer. During the electrochemical deposition process, zinc ions travel through the water layer to reach the zinc substrate surface in both bare zinc (Bare Zn) and OIL-IPS modified zinc (OIL-IPS@Zn) systems. The free energy is continuously monitored during the zinc-ion transportation through the surface layer. The highest energy barrier is recorded as the transportation resistance.

## Results and Discussion

### Design of the Immobilized Zwitterionic Molecular Brush in Functional OIL on the Zn Metal Anode

**Fig. 1:**
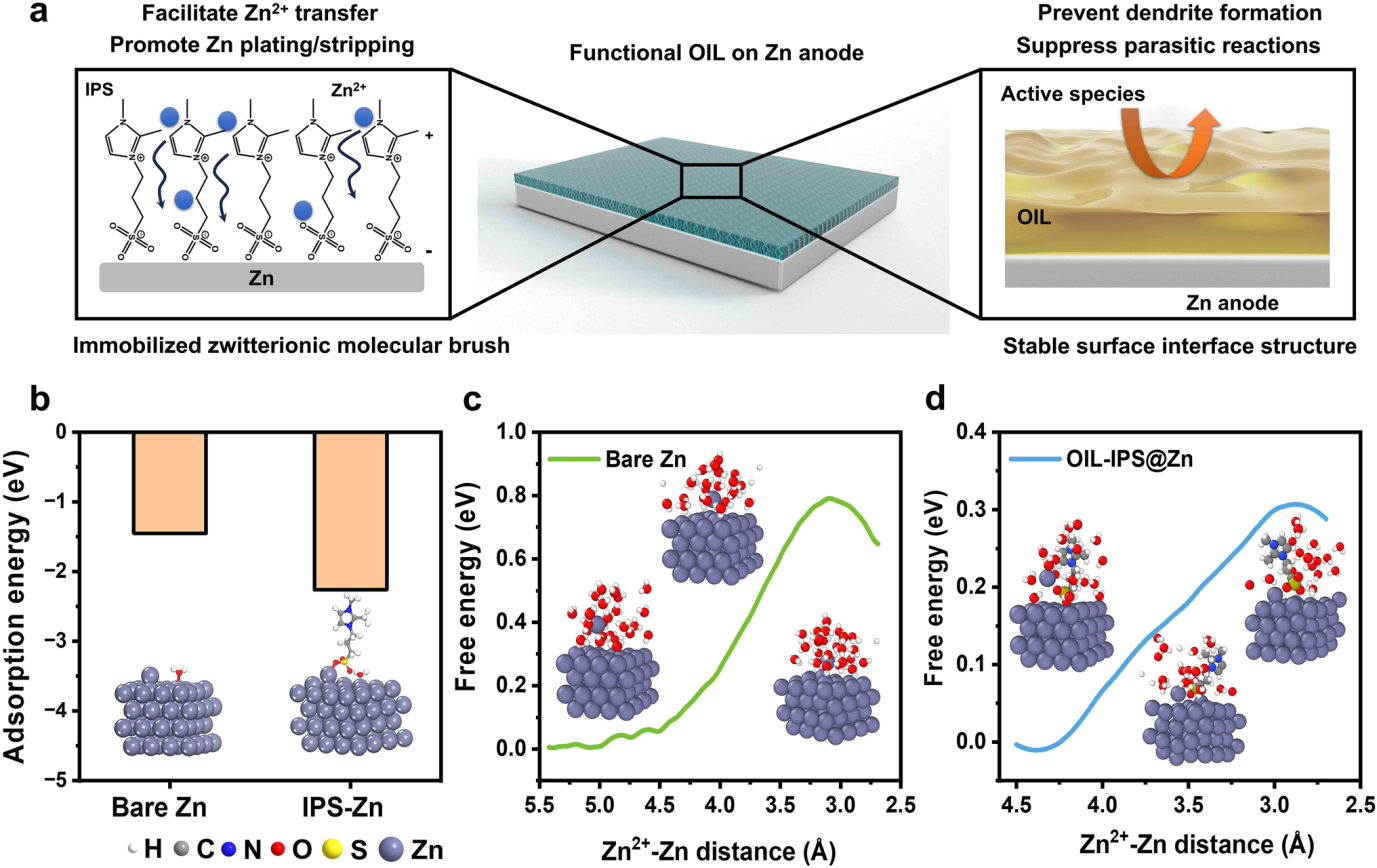
Theoretical prediction of functional OIL to improve the properties of Zn anodes. **a** Schematic illustration of OIL structures from the OAPC process and their functional mechanisms. **b** DFT calculated adsorption energy of Zn atoms on the Bare Zn and the OIL-IPS@Zn anodes. MD simulated the passage of Zn^2+^ through the SEI layer on the surface of **c** Bare Zn and **d** OIL-IPS@Zn

Figures 1a and S1 illustrate the strategy to address the challenges of dendrite growth and parasitic reactions on Zn metal anodes. Two possible solutions are highly beneficial: facilitating fast Zn plating and stripping, and constructing stable surface structures. The construction of a densely packed surface protection layer with stable electric double layers satisfies both criteria. A zwitterionic molecule IPS is introduced to the surface of Zn to construct the artificial electric double layer (characterizations in Figs. S2–S5 and Table S1). Through performing DFT calculations, the adsorption energy of the Zn^2+^ on the zwitterion IPS-tethered surface shows a significantly lower value of − 2.26 eV than that on the bare Zn (− 1.45 eV) (Fig. 1b). This is owing to the capability of the zwitterion IPS layer to re-distribute the surface charge, which can facilitate the adsorption and conversion of Zn^2+^, leading to a smooth Zn plating and stripping [47–49]. Furthermore, molecular dynamic (MD) simulations on the anodes also reveal that Zn^2+^ is much easier to transfer to the surface of Zn metal with the aid of surface-tethered zwitterion IPS compared to the bare Zn metal anode (Fig. 1c, d). The transportation resistance is significantly reduced, verified by the reduced energy barriers for the transition states (0.30 vs. 0.79 eV, respectively). As a result, incorporating zwitterionic IPS to construct artificial electric double layers should be highly beneficial to the Zn plating and stripping.

The direct application of zwitterionic IPS in the aqueous electrolytes to *in situ* generate artificial electric double layers, however, could not resolve the stability issues. The SEI generated from the aqueous electrolyte consists of abundant water molecules, which tend to have aqueous-solvent-derived loose and porous SEI due to continuous hydrogen evolution reaction (HER). This could lead to the interruption of the artificial electric double layers and the gradual dissolution of the IPS molecules. Organic-solvent-derived SEI, on the other hand, often shows densely packed morphology compared to the aqueous-derived one, making it suitable for immobilizing the zwitterionic IPS layers while maintaining a stable protection layer on the Zn anode (Fig. S6). However, the pure organic-solvent-derived SEI may not be fully compatible with aqueous electrolytes, which may negatively influence the electrochemical properties of Zn anodes in the aqueous electrolyte. Therefore, exploring the construction of such layers in the mixture condition may be the most viable strategy.

**Fig. 2:**
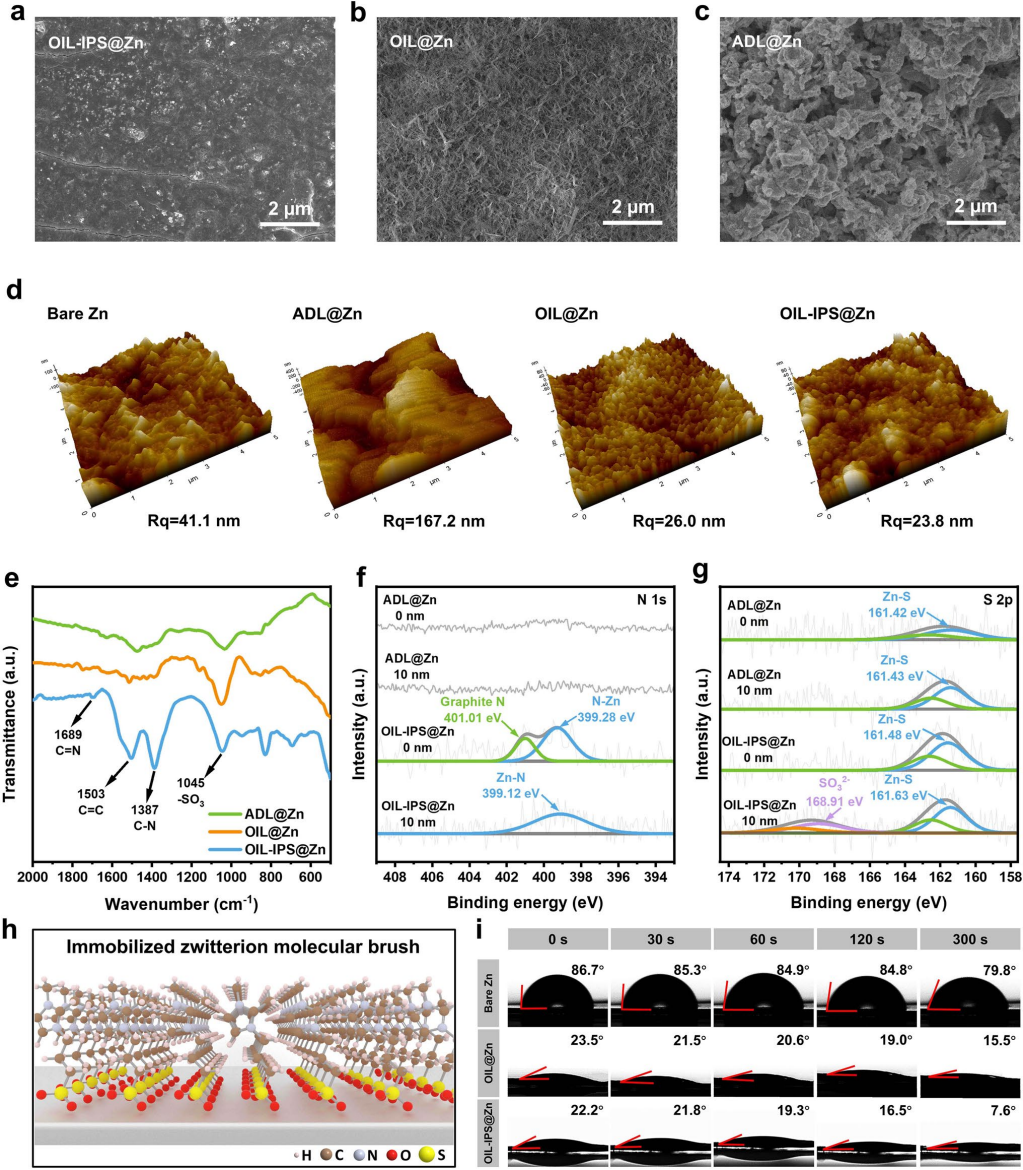
Characterizations of functional OIL on Zn metal anodes. SEM images of **a** OIL-IPS@Zn, **b** OIL@Zn, **c** ADL@Zn. **d** AFM images of Bare Zn, ADL@Zn, OIL@Zn and OIL-IPS@Zn electrodes. **e** FTIR spectrum of ADL@Zn, OIL@Zn and OIL-IPS@Zn anodes. OIL-IPS@Zn, and ADL@Zn anode spectra of **f** N 1*s*, and **g** S 2*p* XPS at different depths. **h** Schematic of IPS molecular brush in SEI at OIL-IPS@Zn. **i** The contact angle between three electrodes and the electrolyte

Guided by the theoretical prediction, the functional OIL with the immobilized zwitterionic molecular brush can be constructed on the surface of Zn metal anodes (OIL-IPS@Zn) through a well-designed OAPC process, which typically refers to pre-treating Zn anodes in organic-water-mixed electrolytes with IPS additives. The optimal conditions for the OAPC process have been explored to construct the functional OIL with the most suitable properties, ensuring smooth Zn anode behavior with high stability. The OIL-IPS@Zn obtained from 0.5 M Zn(OTf)_2_ in a propylene carbonate and water mixture (volume ratio 3:2) with 50 mM IPS is identified as displaying the optimal electrochemical performance (Figs. S7–10). The functional OIL from the OAPC process displays optimal properties. Comparison samples were also prepared, including OIL@Zn with a similar OAPC process without the addition of IPS molecules, and Zn with aqueous-derived layers (ADL@Zn) treated with a similar condition in the aqueous solution instead of organic ones. Compared to the uneven morphology of ADL@Zn (Fig. 2c), the Zn metal from the OAPC process displays a homogeneous architecture coverage, indicating the successful formation of densely packed layers on the surface (Fig. 2a). It appears that the addition of zwitterionic IPS has negligible effects on the SEI morphology (Figs. 2b and S11–S13), indicating the densely packed structure originates from the organic solvent environment during the OAPC process. Atomic force microscopy (AFM) in Figs. 2d and S14 show that both OIL@Zn and OIL-IPS@Zn exhibit relatively dense and smooth surfaces with small root mean square roughness (Rq) of 26.0 and 23.8 nm, respectively, which is even smaller than the untreated bare Zn surface of 41.1 nm. In contrast, ADL@Zn shows a significant increase in Rq of over 167.1 nm, referring to the dendrite formation in aqueous electrolytes with no protection. Such densely packed surface layer will display excellent resistance to parasitic reactions, fostering a stable environment for long-term Zn plating/stripping in the Zn-ion batteries. Furthermore, the OIL with the stable structure can also create confined anchoring sites for IPS packs, immobilizing the organic molecules and stabilizing the functional structures during the battery reactions. FTIR spectroscopy performed on the OIL-IPS@Zn reveals the distinguishable peaks of IPS (Figs. 2e, S15 and S16), the characteristic peak of OIL-IPS@Zn at 1045, 1387, 1503, and 1689 cm^−1^ are reasonably attributed to the IPS, and peaks appear at 1689 and 1387 cm^−1^corresponding to the C=N and C–N bond of IPS. These peaks are absent in OIL@Zn and ADL@Zn, indicating the successful insertion of IPS in the functional OIL [50–53]. The energy dispersive spectrometer (EDS) on the OIL-IPS@Zn displays a homogeneous distribution of IPS molecules in the functional OIL (Fig. S17 and Table S2) [54]. The XAS spectra of the pre-treated electrodes also indicate the existence of IPS on the surface of OIL-IPS@Zn, forming interactive bonds to the Zn metal (Fig. S18). Compared to the ADL@Zn, the results of XPS measurements coupled with Ar^+^ sputtering display the presence of the Graphite N (401.01 eV) on the surface of OIL-IPS@Zn and the SO_3_^2−^ (168.91 eV) at the depth of 10 nm of OIL-IPS@Zn corresponding to the imidazolium structure and sulfuric groups in the functional OIL, respectively, which is consistent with the arrangement of IPS molecules in SEI, demonstrating the successful insertion of the IPS molecules in the functional OIL on the OIL-IPS@Zn (Fig. 2f) [55]. It is worth mentioning that the imidazolium signal has been primarily found on the top of the interfacial layer, which refers to the gathering of the imidazolium cations at the outer side of the organic interfacial layer (Fig. 2f). The opposite trend observed for the S element related to SO_3_^2−^ indicates that the majority is present on the inner side of the functional OIL, suggesting that the sulfuric groups of IPS tend to localize at the bottom of the interfacial layer. (Figs. 2g, S19–S21 and Table S3). This shows that the zwitterion IPS has not only been inserted and immobilized in the functional OIL, but also arranged in a uniform orientation in that cation groups are close to the electrolyte, while the anion groups are grafted to the Zn surface, forming the immobilized zwitterionic molecular brush in the functional OIL on the Zn anode (Fig. 2h). This phenomenon originates from the directional movement of the IPS molecules during the OAPC process under the influence of electric field. The unique immobilized zwitterionic molecular brush layer consisting of IPS molecules can function as the artificial electric double layer contributing to charge re-distribution and smooth Zn plating/stripping, while the packed brush structure in the densely packed interfacial layers can also prevent direct contact between Zn metal anodes and other active species from the electrolyte, leading to the suppression of parasitic reactions. Subsequently, TOF–SIMS result on the OIL-IPS@Zn (Fig. S22) shows a similar trend to the XPS, which further verifies the formation of molecular brush structure in the SEI. FTIR and XPS measurements on the anodes after long-term cycling further confirm the stability of the functional OIL on the Zn anode. As shown in Fig. S23, the FTIR spectra of the OIL-IPS@Zn display negligible change before and after cycling with the characteristic peaks of IPS. Meanwhile, the XPS results reveal that the structure of the molecular brush is unchanged, verifying the stability of the functional layer (Fig. S23 and Table S4).

Contact angle measurements on these pre-treated Zn metal anodes (Figs. 2i and S24–S26) verify the affinity of the functional OIL to the aqueous electrolytes (0.5 M Zn(OTf)_2_) for Zn-ion batteries. The untreated bare Zn anode shows a moderate wettability of the electrolyte, displaying a contact angle of 86.7°. In contrast, the anodes from the OAPC process exhibit much lower contact angles compared to the bare ones, suggesting excellent hydrophilicity of these densely packed interfacial layers. Such wettability will significantly promote smooth Zn plating and stripping by increasing the contact area between the electrode and electrolyte while facilitating the ion transport migrating through the interfacial layers [27]. It is worth noting that the Zn anode from aqueous pre-treatment displays a reduced contact angle (30.6°) compared to the bare Zn, which is still much higher than those from the OAPC process, indicating the organic-assisted interlayer formation has a better affinity toward electrolytes than the aqueous-assisted process (Figs. S27 and S28). Furthermore, OIL-IPS@Zn shows better wettability than OIL@Zn, owing to the unique immobilized zwitterionic molecular brush structure with intrinsic hydrophilicity, which leads to a better permeability of the aqueous electrolytes [56]. Intriguingly, both Zn anodes from the OAPC process display a gradual decrease in contact angles with time while no visible change is observed in the aqueous-assisted anodes. This is because the densely packed interfacial layer consists of fragments originating from both organic and aqueous solvents. The synergistic effects between these fragments increase the wettability, allowing the electrolyte droplets quickly to spread through the surface. Additionally, OIL-IPS@Zn features a high content of hydrophilic IPS molecules in the immobilized zwitterionic molecular brush, displaying a faster decline in the contact angle, which will significantly benefit the electrochemical performance of Zn plating/stripping.

### Electrochemical Characterization of Zn Anode with Functional OIL

**Fig. 3:**
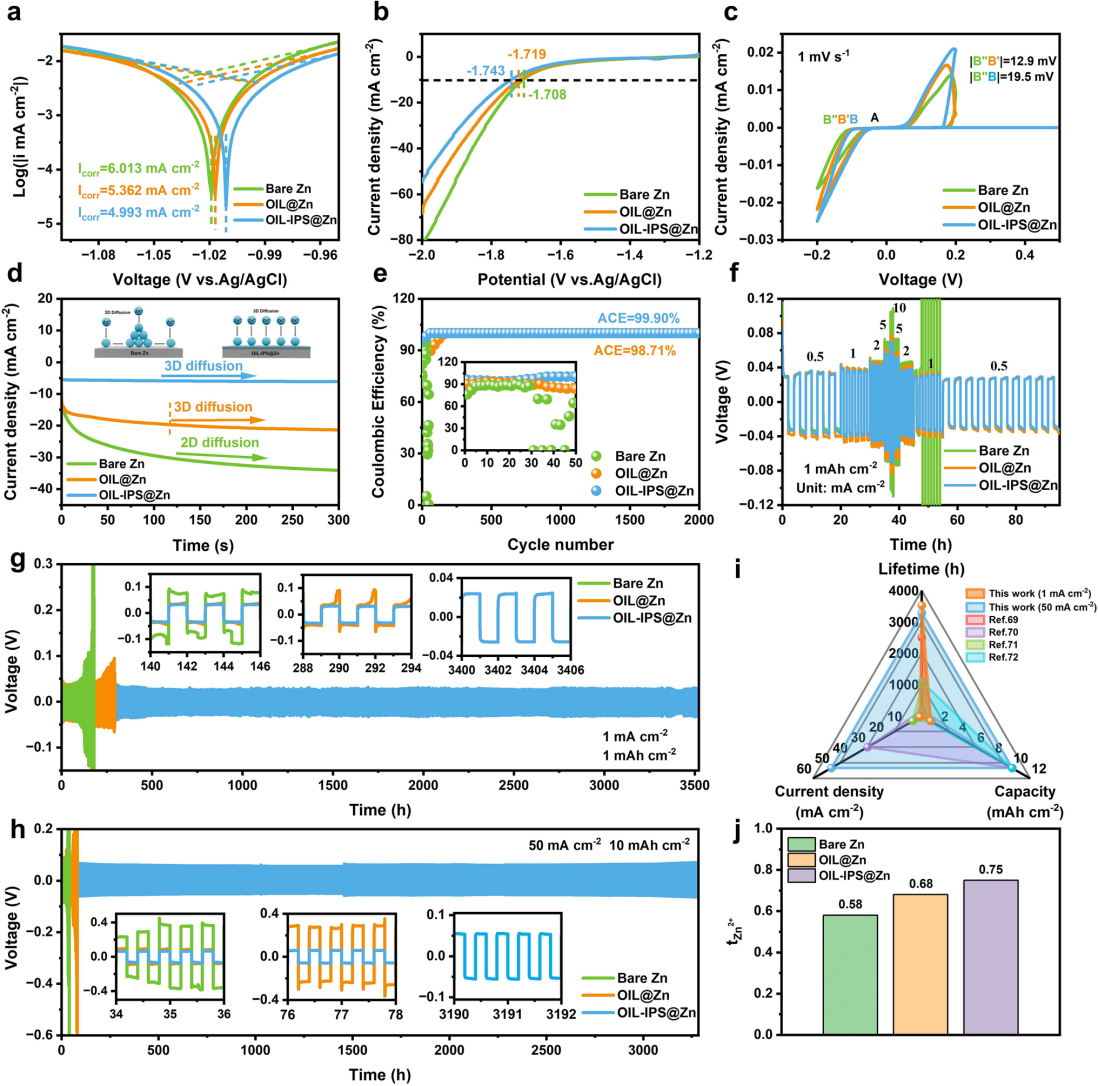
Electrochemical performance test of OIL-IPS@Zn electrodes. **a** Tafel plots, **b** LSV curves, and **d** CA curves that were tested in three-electrode systems. **c** CV curves of Zn||Cu batteries at a scan rate of 1 mV s^−1^. **e** Comparison of CE between Bare Zn||Cu, OIL@Zn||Cu and OIL-IPS@Zn||Cu half cells during cycles at 1 mA cm^−2^ with an areal capacity of 1 mAh cm^−2^. **f** Rate capability of symmetrical cells assembled using Bare Zn, OIL@Zn and OIL-IPS@Zn anodes at various current densities from 0.5 to 10 mA cm^−2^. Long-term galvanostatic cycling of symmetric Zn cells with Bare Zn, OIL@Zn and OIL-IPS@Zn electrode at **g** 1 mA cm^−2^/1 mAh cm^−2^, and **h** 50 mA cm^−2^/10 mAh cm^−2^. **i** Comparison of the performance of our symmetric cells at different current densities with previously reported results. **j** Zn^2+^ transference number

Tafel curves in Fig. 3a demonstrate the corrosion resistance and corrosion rate of the treated anodes in the aqueous electrolyte [57]. The corrosion potential of the OIL-IPS@Zn anode is −1.008 V (vs Ag/AgCl), while that of the OIL@Zn anode is −1.018 V (vs Ag/AgCl), both of which are lower than the bare Zn electrode (− 1.023 V vs Ag/AgCl). Furthermore, OIL-IPS@Zn shows the lowest corrosion current density of 4.993 mA cm^−2^, compared to the bare Zn electrode (6.013 mA cm^−2^) and the OIL@Zn (5.362 mA cm^−2^), demonstrating a strong resistance of OIL-IPS@Zn against corrosion [31, 58]. It can be identified that the OIL-IPS@Zn anode effectively suppresses the HER, through linear sweep voltammetry (LSV) in a Na_2_SO_4_ electrolyte (Fig. 3b). OIL-IPS@Zn displays a much higher onset potential than OIL@Zn and bare Zn anode, referring to a lower possibility of HER during the Zn battery reaction. At 10 mA cm^−2^, OIL-IPS@Zn shows a high potential of − 1.743 V, compared to OIL@Zn (− 1.719 V) and Bare Zn (− 1.708 V). This clearly suggests that the unique structure of functional OIL with the immobilized zwitterionic molecular brush structure on the Zn metal anode can efficiently suppress HER while providing better stability at a wider potential window [58]. Cyclic voltammetry (CV) was conducted to evaluate the nucleation potential of Zn||Cu cells. Comparing the nucleation overpotential (NOP) of Bare Zn, OIL@Zn, and OIL-IPS@Zn, it is observed that the NOP of OIL@Zn and OIL-IPS@Zn are below the value required for deposition of Bare Zn (|B′′B′|= 12.9 mV, |B′′B|= 19.5 mV), which is attributed to the nucleation of Zn^2+^, indicating that the functional OIL with immobilized zwitterionic molecular brush structure facilitates nucleation with a lower energy barrier owing to the charge re-distribution (Figs. 3c, S29 and S30) [59]. Subsequently, this leads to uniform deposition field distribution on the OIL-IPS@Zn electrode, which is highly beneficial for Zn stripping/plating. Chronoamperometry (CA) identifies the Zn plating mechanism on these Zn anodes (Fig. 3d). The current density of the bare Zn electrode quickly exceeded 34.12 mA cm^−2^ within 300 s, indicating the dominant two-dimensional plane diffusion of Zn^2+^ occurred on the bare Zn electrode. In contrast, both OIL@Zn and OIL-IPS@Zn reveal a significant decrease in the current densities, reaching 21.37 and 6.15 mA cm^−2^, respectively, within the same time. Meanwhile, both show a stable curve which refers to a typical three-dimensional diffusion on the anode surface. It is generally known that two-dimensional diffusion along the surface is disadvantageous as it leads to dendritic growth at the tips, while Zn^2+^ can be locally reduced to Zn^0^ in three-dimensional diffusion, thereby inhibiting dendritic growth [60–62]. The stable Zn plating/stripping on OIL-IPS@Zn is achieved by assembling Zn||Cu half cells, which shows a CE of 99.90% over 2000 cycles (Figs. 3e and S31). This is significantly higher than the bare Zn electrode, which exhibited unstable plating/stripping behavior with only 30 cycles operation [63, 64]. It verifies that the densely packed functional OIL has the capability to transfer Zn^2+^ while preventing parasitic reactions, leading to a smooth Zn plating/stripping process. The immobilized zwitterionic molecular brush structure also plays an important role, as slightly lower CE was found with the OIL@Zn||Cu half cells (98.71%), indicating such structure increases the affiliation toward electrolytes and facilitates stable Zn^2+^ transfer and deposition. We further constructed the symmetrical Zn||Zn cells to evaluate their capability as a stable anode for Zn-ion batteries. Their tolerance was examined in different current densities by conducting the rate performance of these anodes. As shown in Fig. 3f, OIL-IPS@Zn displays a stable performance at a wide current density range from 0.5 to 10 mA cm^−2^. Furthermore, the OIL-IPS@Zn also possesses excellent retention when the current reverts back to 0.5 mA cm^−2^, demonstrating its capability as a potential stable anode for Zn- ion batteries. OIL@Zn shows similar performance except for higher potential with slightly lower retention capability, due to the lack of the artificial electric double layer for stable Zn plating/stripping. In contrast, the bare Zn symmetrical cell experienced short circuit at large current densities (Figs. S32 and S33) [65, 66]. The electrochemical cycling performance of these anodes was evaluated at 1 mA cm^−2^ and 1 mAh cm^−2^ (Figs. 3g, S34 and S35). The bare Zn||Zn symmetrical cell failed around 160 h due to the accumulation of parasitic products and the formation of Zn dendrites [67, 68]. In contrast, both Zn anodes from the OAPC process showed better cyclic capability. Notably, the performance of OIL@Zn remains stable for over 216 h, while the OIL-IPS@Zn exhibited remarkable cycling stability for over 3500 h. EIS measurement was conducted on the symmetrical cells before and after long-term cycling (Fig. S36). The results indicate that the impedance of OIL-IPS@Zn remain stable after long-term cycling, verifying the stability of the anode with suppressed dendrite formation. Moreover, the OIL-IPS@Zn||OIL-IPS@Zn symmetrical cell could operate stably for over 3200 h at a current density of 50 mA cm^−2^ and a capacity density of 10 mAh cm^−2^, while the comparison anodes could only last for 32 h (bare Zn) and 58 h (OIL@Zn) under the same conditions (Fig. 3h). These results clearly demonstrate that the immobilized zwitterionic molecular brush structure in functional OIL from the OAPC process can efficiently facilitate the Zn stripping/plating by enhancing Zn^2+^ transportation while preventing the formation of parasitic products and dendrites (Fig. S37), leading to excellent performance that is comparable to most published references (Fig. 3i and Table S5) [69–72]. In addition, the electrochemical results of the symmetric Zn cell with high DOD (Fig. S38) indicate that even at high DOD, the OIL-IPS@Zn symmetric cell can maintain an excellent cycling capability. This is due to the excellent properties of functional OIL on the Zn anode which provides excellent ionic conductivity, facilitates Zn plate/stripping on the electrode surface below the functional OIL, and suppresses dendrite growth, leading to the stable operation of the symmetric cell. Furthermore, the transfer number of Zn^2+^ (t_Zn2+_) is determined by EIS and chronoamperometry at a constant polarization voltage of 10 mV in symmetric cells of Bare Zn, OIL@Zn and OIL-IPS@Zn (Figs. 3j and S39). The result shows that the t_Zn2+_ of bare Zn||Zn is 0.58, which is due to the application of a non-negligible Zn^2+^ concentration gradient near the Zn electrode and the formation of a strong interfacial polarization [73, 74]. On the contrary, the t_Zn2+_ of OIL@Zn||OIL@Zn and OIL-IPS@Zn||OIL-IPS@Zn increase significantly to 0.68 and 0.75, indicating that the functional OIL is conducive to the transport of Zn^2+^.

### Postmortem Characterizations for Mechanism Research

**Fig. 4:**
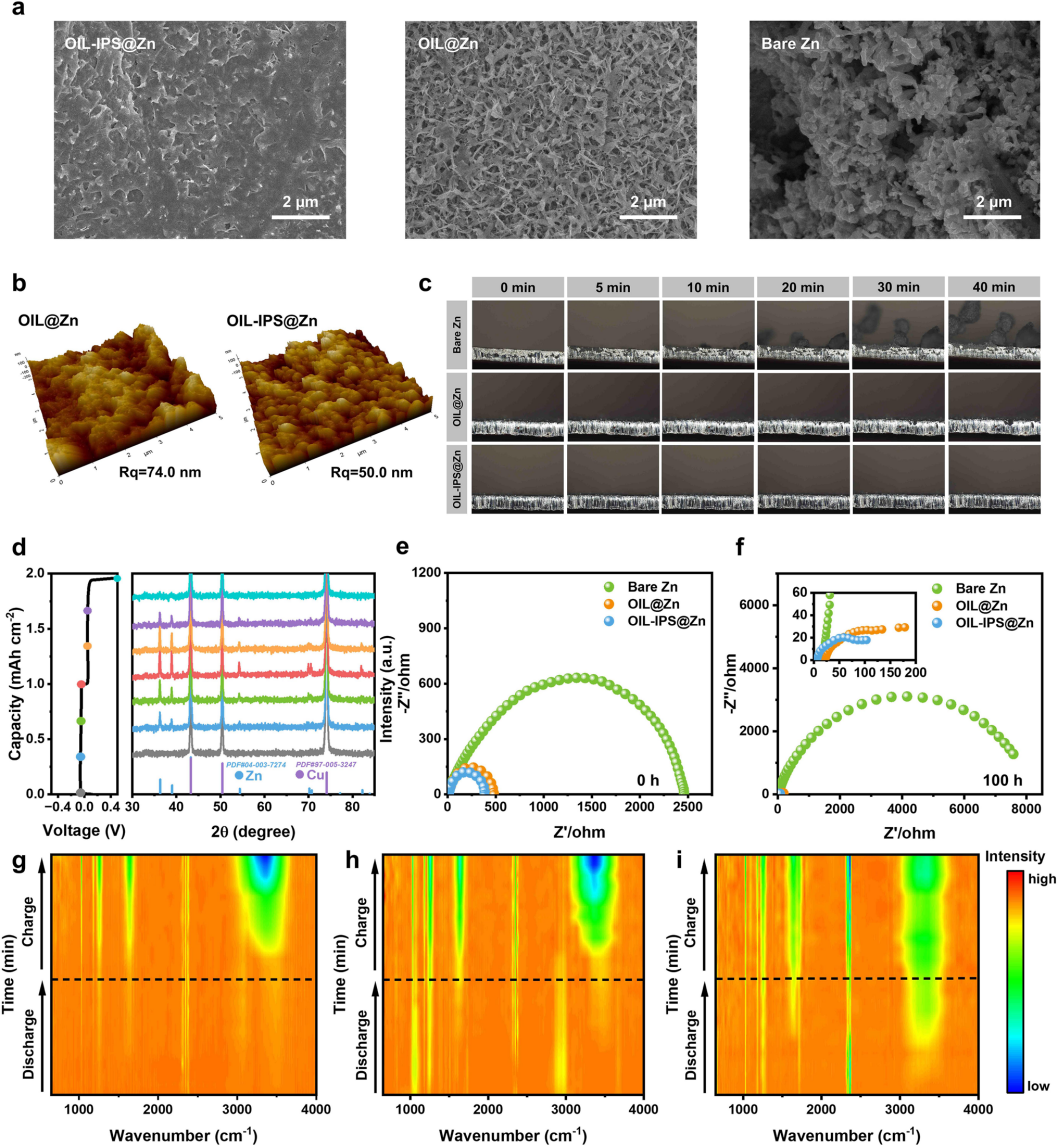
Postmortem and in situ characterizations of Zn surface with functional OIL. **a** SEM images of Bare Zn, OIL@Zn and OIL-IPS@Zn electrodes deposited at 1 mA cm^−2^, 1 mAh cm^−2^ for 10 cycles. **b** AFM images of OIL@Zn and OIL-IPS@Zn electrodes deposited at 1 mA cm^−2^, 1 mAh cm^−2^ after 14 h. **c** Bare Zn, OIL@Zn and OIL-IPS@Zn electrodes in symmetric cells at 5 mA cm^−2^ for 40 min. **d** Ex situ XRD pattern of the Cu electrode during the first Zn plating/stripping cycle. EIS images of Bare Zn, OIL@Zn and OIL-IPS@Zn symmetric batteries cycling for **e** 0 h and **f** 100 h. In situ ATR-SEIRAS results of **g** Bare Zn, **h** OIL@Zn and **i** OIL-IPS@Zn electrode symmetrical cells

Postmortem characterizations (Figs. 4a and S40–S42) of these anodes after cycling reveal the functional mechanisms responsible for the excellent capability of the functional OIL in enhancing the stability of Zn anodes. It is clear that the bare Zn anodes display an irregular shape surface morphology, referring to the formation of unstable SEI layers and dendrites with the accumulation of parasitic products. Both anodes after the OAPC process show a smooth surface after cycling, indicating the excellent capability of the organic interfacial layer to prevent dendrite formation. In particular, the OIL-IPS@Zn exhibits a much denser surface structure than OIL@Zn. Similar results were found for the AFM characterization that OIL-IPS@Zn reveals a much smaller Rq than the bare Zn and OIL@Zn. Compared to the original Rq in Fig. 4b, a small increase of 26.2 nm is found on OIL-IPS@Zn after cycling, while the bare Zn anode shows a significant increase of 126.1 nm (Figs. 2d and S14), indicating a stable interface is present on the surface of OIL-IPS@Zn (Fig. S43). These results verify that IPS molecules can not only modulate the double layer, but also contribute to stable SEI formation. We further performed in situ optical microscopy to observe the Zn deposition process in the aqueous electrolyte to verify the Zn plating/stripping on these anodes. As shown in Figs. 4c, S44 and S45, obvious dendrite growth is spotted on the bare Zn electrode just after 10 min, and the uncontrolled growth of Zn dendrites occurs with time, reaching approximately 300 μm after 40 min of deposition. OIL@Zn shows a better Zn deposition within 30 min compared to the bare Zn anode, owing to the stable organic interfacial layer. However, dendrite growth could be spotted after 30 min of plating. In contrast, OIL-IPS@Zn exhibited a smooth Zn plating, and no visual dendrite growth was spotted during the entire plating process [75]. This observation is verified by ex situ XRD characterizations performed at different discharge and charge stages (Figs. 4d and S46) that the signal of Zn metal appears and intensifies during discharge, while during charge, the intensity of Zn metal gradually decreases until it completely disappears at the end of charge.

Such smooth performance confirms the reversibility of the plating and stripping processes and also indicates the absence of parasitic product formation during this process, allowing for the potential long-term operation of Zn anode in Zn-ion batteries. The excellent properties originate from the stable functional OIL, featuring an immobilized zwitterionic molecular brush structure formed through the OAPC process. The densely packed organic layer can easily prevent the long-term exposure of the bare Zn to the electrolytes to cause parasitic reactions, while the charge re-distribution in the zwitterion IPS molecule layers facilitates the Zn^2+^ transportation, enabling smooth Zn plating and stripping (demonstrated by EIS in Fig. 4e, f), leading to smooth Zn plating and stripping on the anodes. As shown in EIS, both OIL@Zn and OIL-IPS@Zn show significant decrease in the resistance, while OIL-IPS@Zn displays the highest conductivity (Fig. S47). In situ FTIR monitors the dynamic changes in the chemical species on the interface during discharge and charge processes. As shown in Figs. 4g–i and S48, the intensities of free water (–OH at 3300 cm^−1^) increase for all three anodes during charge, which refers to the desolvation of Zn^2+^ and the repulsion of the dissociated water molecules. Interestingly, the intensity of –OH on the OIL-IPS@Zn electrode maintains a high value even during the discharge process, which indicates the excellent affiliation of artificial electric double layers to keep water molecules on the surface, leading to facilitating the Zn stripping during the discharge process [76–78]. This also demonstrates the important role of immobilized zwitterionic molecular brush structure in functional OIL to enhance electrochemical performance.

### Electrochemical Performance of full Zn-Ion Batteries

It is demonstrated that OIL-IPS@Zn, with the artificial functional OIL, can perform smooth Zn plating and stripping with high stability, making it a potential candidate for full Zn-ion batteries. Therefore, we assembled the full aqueous zinc-ion battery (AZIB) using the OIL-IPS@Zn as the anode, H_2_V_3_O_8_-based materials as the cathode, and 0.5 M Zn(OTf)_2_ as the electrolyte to evaluate their potential practical applications [79–81].

**Fig. 5:**
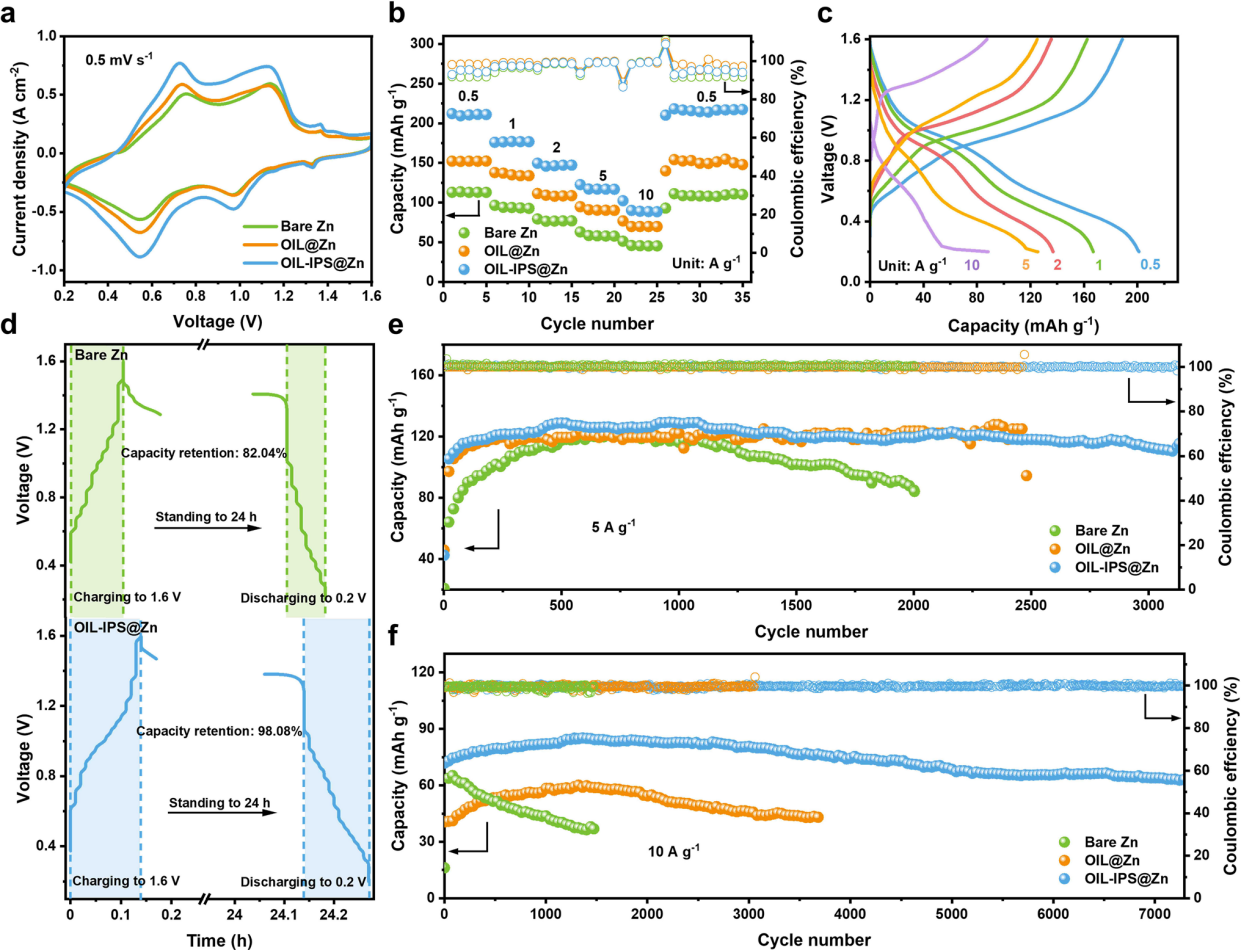
Electrochemical performance of full cell batteries. Comparison of Bare Zn||H_2_V_3_O_8_, OIL@Zn||H_2_V_3_O_8_ and OIL-IPS@Zn||H_2_V_3_O_8_ full cells: **a** CV curves with a scan rate of 0.5 mV s^−1^, **b** rate performance. **c** Galvanostatic charge–discharge curves of the OIL-IPS@Zn||H_2_V_3_O_8_ coin cell at different rates. **d** Comparison of self-discharge behavior of Bare Zn, and OIL-IPS@Zn. The cells were subjected to charging at 5 A g^−1^ to 1.6 V, standing for 24 h, and then discharging to 0.2 V. Cycling stability **e** at 5 A g^−1^, and **f** at 10 A g^−1^

H_2_V_3_O_8_ nanowires were synthesized through a hydrothermal method (Fig. S49 and Table S2). The CV curves in Fig. 5a show two reversible pairs of oxidation/reduction peaks corresponding to the multi-step redox couples of V^3+^/V^4+^ and V^4+^/V^5+^ [82, 83]. Compared to the cells with bare Zn anode, those with OIL@Zn and OIL-IPS@Zn exhibited much higher current responses, which can be attributed to faster Zn plating and stripping efficiency [84, 85]. OIL-IPS@Zn shows the highest current densities for these redox peaks, indicating the improved Zn^2+^ migration rate due to the artificial electric double layer. Additionally, the AZIB with OIL-IPS@Zn exhibited excellent rate performance, showing excellent cycling stability at various current densities ranging from 0.5 to 10 A g^−1^ and a remarkable capacity retention when the current density was reversed back to 0.5 A g^−1^ (Figs. 5b, c and S50). In contrast, both batteries with bare Zn and OIL@Zn anodes show a decreased capacity throughout the entire current range, further verifying the excellent capability of OIL-IPS@Zn to support smooth Zn plating and stripping [86, 87]. Figure 5d shows self-discharging capability by resting the cells for 24 h between the discharge and charge processes. The OIL-IPS@Zn||H_2_V_3_O_8_ AZIB retained 98.08% of its initial capacity even after 24 h of rest, while the OIL@Zn||H_2_V_3_O_8_ full cell retained 95.89% of its initial capacity (Fig. S51). In contrast, the Bare Zn||H_2_V_3_O_8_ full cell retained only 82.04% of its initial capacity after 24 h of rest, owing to the excellent capability of the organic interfacial layer as a stable interface to reduce parasitic reactions. As a result, the AZIB with OIL-IPS@Zn as the anode demonstrated excellent cycling stability, maintaining an over 91.69% capacity retention after 3130 cycles at 5 A g^−1^ (Fig. 5e), while the OIL@Zn||H_2_V_3_O_8_ AZIB showed stable operate within 2480 cycles, with severe capacity decay afterward. In contrast, the AZIB with bare Zn anodes displayed a rapid capacity decay after 70 cycles, which continued to drop below 85 mAh g^−1^ after 1000 cycles at the same current density, attributing to parasitic reactions and dendrite growth. It is worth noting that the capacity of the full battery increases slowly first 500 cycles of charge and discharge, which is due to the activation stage of the battery, the initial increase in the capacity should be assigned to the activation process of the cathodes. Thus, with the aid of the stable functional OIL, the AZIB with OIL-IPS@Zn as the anode and H_2_V_3_O_8_ as the cathode could maintain outstanding cycling stability for over 7000 cycles at 10 A g^−1^ with excellent capacity retention, while the one with bare Zn anode showed significant decay in the capacity after 60 cycles (Fig. 5f). This indicates the OIL-IPS@Zn anode to be highly effective in suppressing parasitic reactions and promoting reversible Zn^2+^ plating/stripping, thus achieving a safe and long-lasting AZIB, leading to excellent performance that is comparable to most published references (Table S6).

## Conclusions

In summary, we successfully designed a functional OIL on the Zn metal anode through the OAPC process. Featuring the immobilized zwitterionic molecular brush structure from the zwitterion IPS molecules, the synthesized functional OIL showed a dense and robust structure with designed artificial electric double layer, which significantly improves the Zn^2+^ transport while suppressing parasitic reactions and dendrite growth. As a result, the resulting OIL-IPS@Zn showed significantly improved properties relating to the electrolyte affinity and ionic conductivity, leading to the enhancement of the reversibility and cycling behavior of Zn stripping and plating. The symmetric cells equipped with OIL-IPS@Zn exhibited over 3500 h operating capability at 1 mA cm^−2^ and 3200 h at 50 mA cm^−2^ with no visible decay. Consequently, paired with the synthesized H_2_V_3_O_8_ cathode, the resulting OIL-IPS@Zn||H_2_V_3_O_8_ full batteries showed great potential under practical conditions, with excellent stability for continuously operating over 7000 cycles. This study demonstrates the feasibility and effectiveness of designing stable Zn electrodes through interfacial design, which holds broad prospects for application in next-generation rechargeable Zn-ion batteries.

## Supplementary Information

Below is the link to the electronic supplementary material.

Supplementary file1 (DOCX 15259 KB)
