# Supplementary material for: Immobilizing Zwitterionic Molecular Brush in Functional Organic Interfacial Layers for Ultra-Stable Zn-Ion Batteries
Source: Nanomicro Lett. 2025 May 20;17:262. doi: 10.1007/s40820-025-01782-5 (PMC12092924; doi:10.1007/s40820-025-01782-5)
Supplement: Supplementary file 1 — Supplementary file1 (DOCX 15259 KB) [file 40820_2025_1782_MOESM1_ESM.docx]

Supporting Information for

Immobilizing Zwitterionic Molecular Brush in Functional Organic Interfacial Layers for Ultra-Stable Zn-Ion Batteries

Limeng Sun^1, 2, #^, Xianjun Cao^1, 2, #^, Li Gao^1^, Jiayi Li^1^, Chen Qian^1^, Jinhu Wu^1^, Xinming Nie^3, *^, Hong Gao^1^, Peng Huang^4, *^, Yufei Zhao^2, *^, Yong Wang^1^, Jinqiang Zhang^2, *^, Guoxiu Wang^2^, Hao Liu^2, *^

^1^ Joint International Laboratory on Environmental and Energy Frontier Materials, School of Environmental and Chemical Engineering, Shanghai University, Shanghai 200444, P. R. China

^2^ Centre for Clean Energy Technology, Faculty of Science, University of Technology Sydney, Broadway, Sydney, NSW 2007, Australia

^3^ School of Physics and Electronic Engineering, Jiangsu Normal University, Xuzhou, Jiangsu 221116, P. R. China

^4^ School of Chemistry and Materials Science, Jiangsu Normal University, Xuzhou, Jiangsu 221116, P. R. China

# Limeng Sun and Xianjun Caocontributed equally to this work.

*Corresponding authors. E-mail: [nxinming@jsnu.edu.cn](mailto:nxinming@jsnu.edu.cn) (Xinming Nie); [huangpeng@jsnu.edu.cn](mailto:huangpeng@jsnu.edu.cn) (Peng Huang); [yufei-zhao@shu.edu.cn](mailto:yufei-zhao@shu.edu.cn) (Yufei Zhao); [Jinqiang.Zhang@uts.edu.au](mailto:Jinqiang.Zhang@uts.edu.au) (Jinqiang Zhang); [Hao.Liu@uts.edu.au](mailto:Hao.Liu@uts.edu.au) (Hao Liu)

**Supplementary Figures and Tables**

**
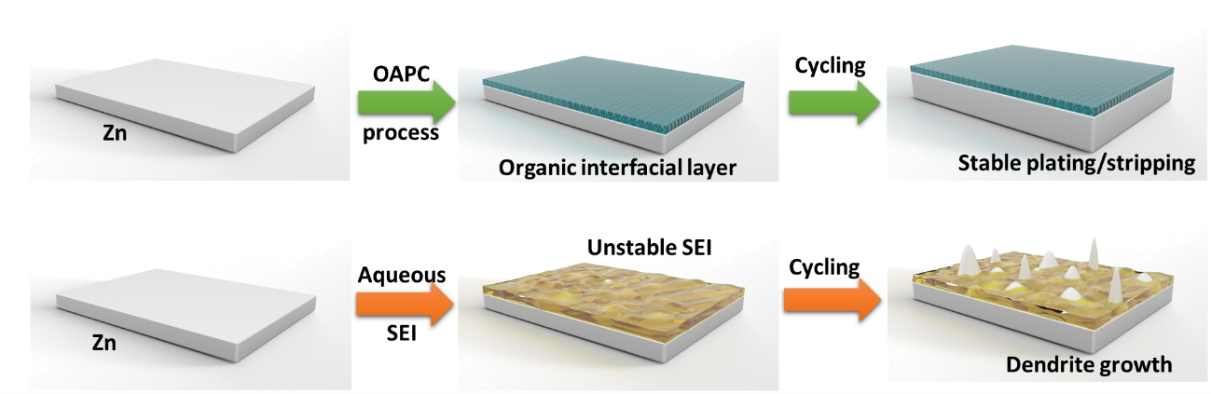
**

**Fig. S1** Schematic illustration of SEI film formation and zinc deposition


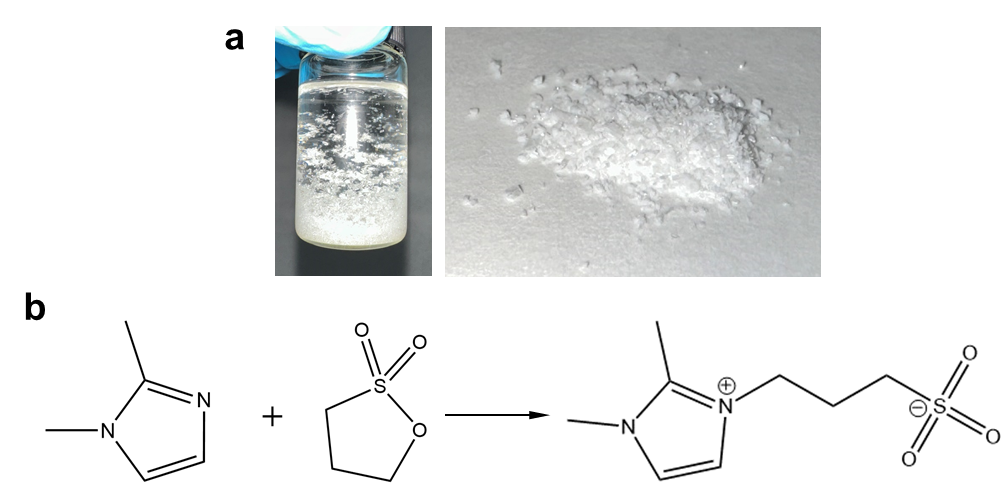


**Fig. S2** (**a**) Digital images, and (**b**) composite diagram of IPS


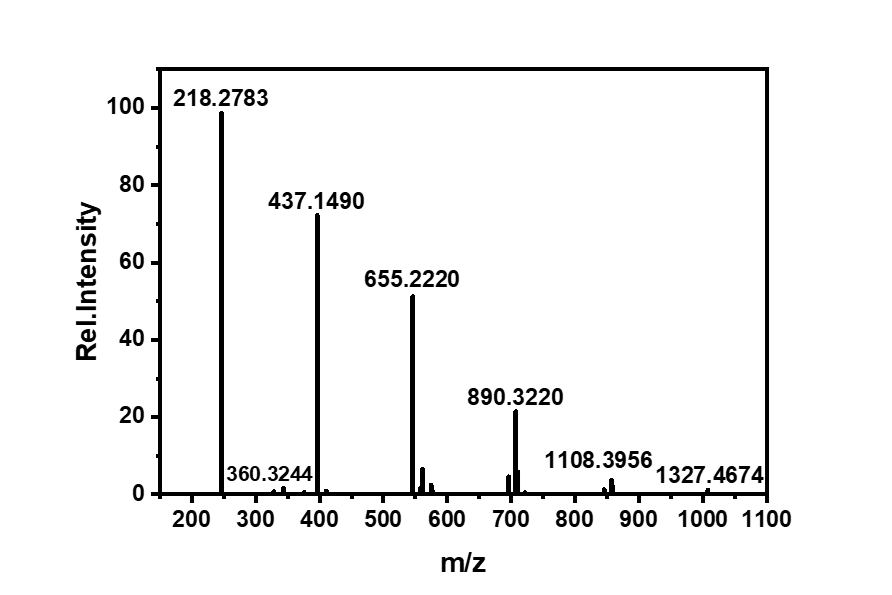


**Fig. S3** ^1^HRMS of IPS

The result shows the IPS structures (molecular weight 218.282) and the aggregates, indicating the success synthesis of IPS molecules.


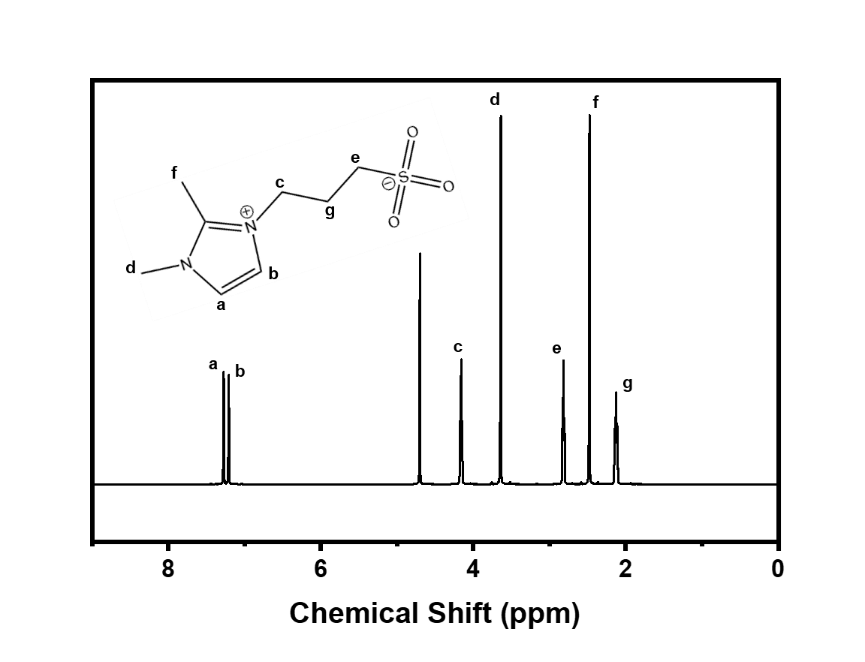


**Fig. S4** ^1^H NMR spectroscopy spectra of 10 ppm IPS. NMR signature peaks (a, b, c, d, e, f and g) show the chemical environment of the hydrogen atoms in IPS, indicating the success synthesis of IPS molecules.


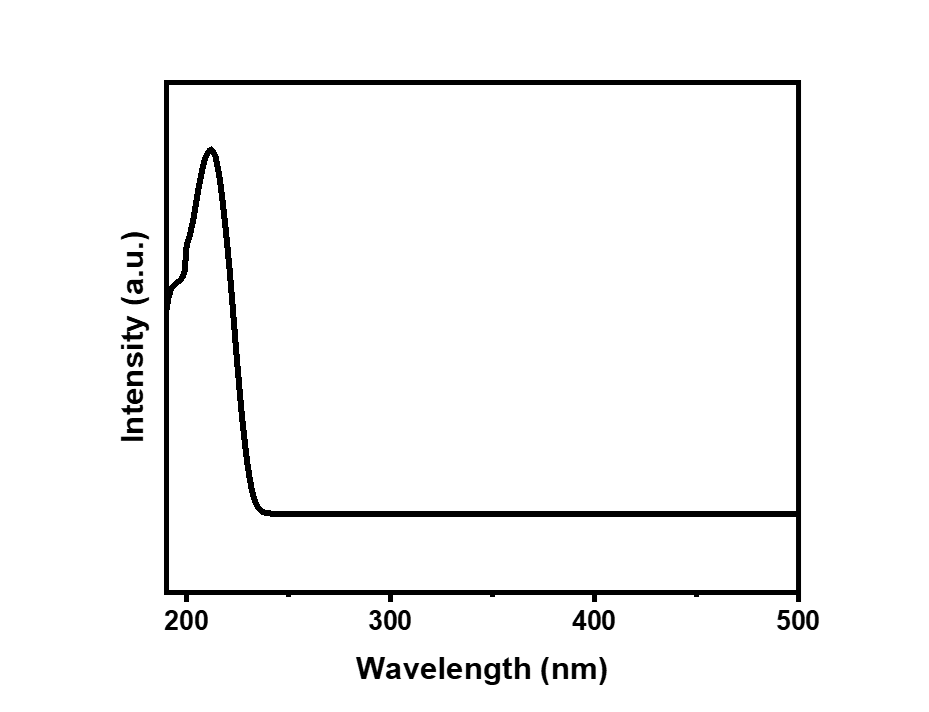


**Fig. S5** Ultraviolet spectra of 100 ppm aqueous solution of IPS.

UV result shows a strong absorption peak at a wavelength of 211.77 nm, which means the presence of two conjugated double bonds in the tested molecule, indicating the successful synthesis of IPS molecules.

**
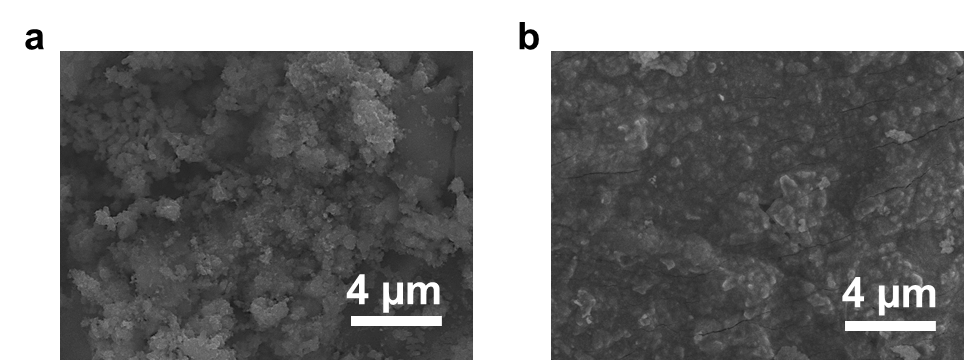
**

**Fig. S6** SEM images of Bare Zn after cycling in (**a**) the aqueous electrolytes, and (**b**) organic electrolyte (near-pure PC solvent)

Since Zn(OTf)_2_ is insoluble in pure PC, we prepared a near-pure PC organic electrolyte by adding 100 μL water and 0.5 M Zn(OTf)_2_ to 5 mL PC.


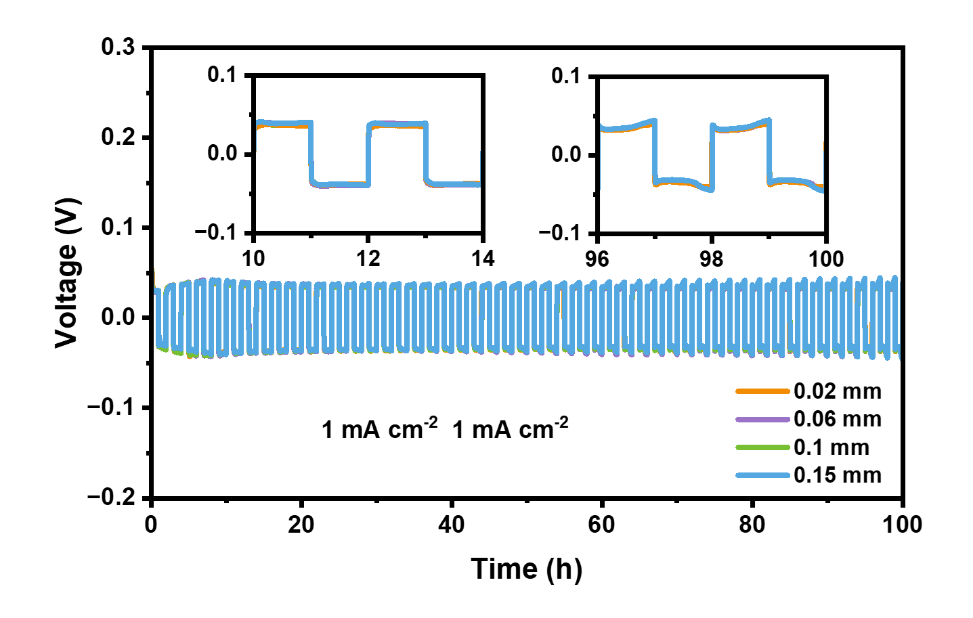


**Fig. S7** Long-term galvanostatic cycling of symmetric Zn cells of the OIL-IPS@Zn anodes using zinc foil with different thicknesses

These cells display similar electrochemical performance, probably because zinc foils with different thicknesses still show excessive Zn for the electrochemical tests, hence no significant differences.


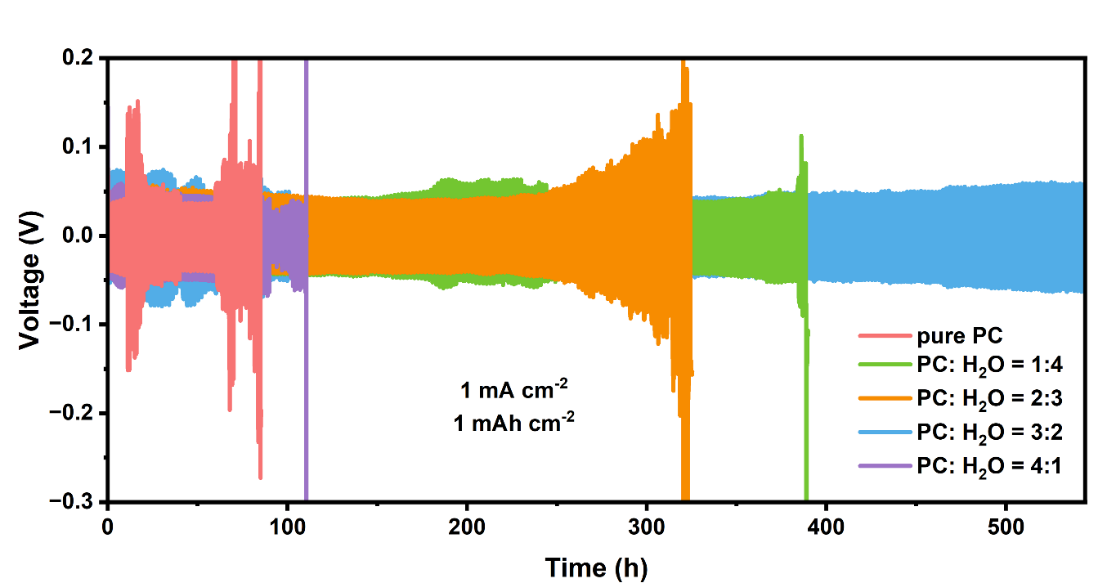


**Fig. S8** Long-term galvanostatic cycling of symmetric cells in aqueous electrolytes with electrodes prepared by OAPC process with electrolytes consisting of different ratios of PC and water.

The electrolytes were prepared by adding 0.5 M Zn(OTf)_2_ to a mixture of PC and water in different ratios (PC: water = 1:4, 2:3, 3:2, and 4:1). Since Zn(OTf)_2_ is insoluble in pure PC,we prepared a near-pure PC organic electrolyte by adding 100 μL water and 0.5 M Zn(OTf)_2_ to 5 mL PC (pure PC).After the OAPC process, the electrodes were assembled into symmetric coin cells with 0.5 M Zn(OTf)_2_ aqueous electrolytes for cycling test. As shown in Fig. S8, the Zn electrode obtained from the OAPC process with the electrolyte (PC: water = 3:2) displays the highest cycling life, thus marking the electrolyte (PC: water = 3:2) best candidate for the follow-up experiments and tests.


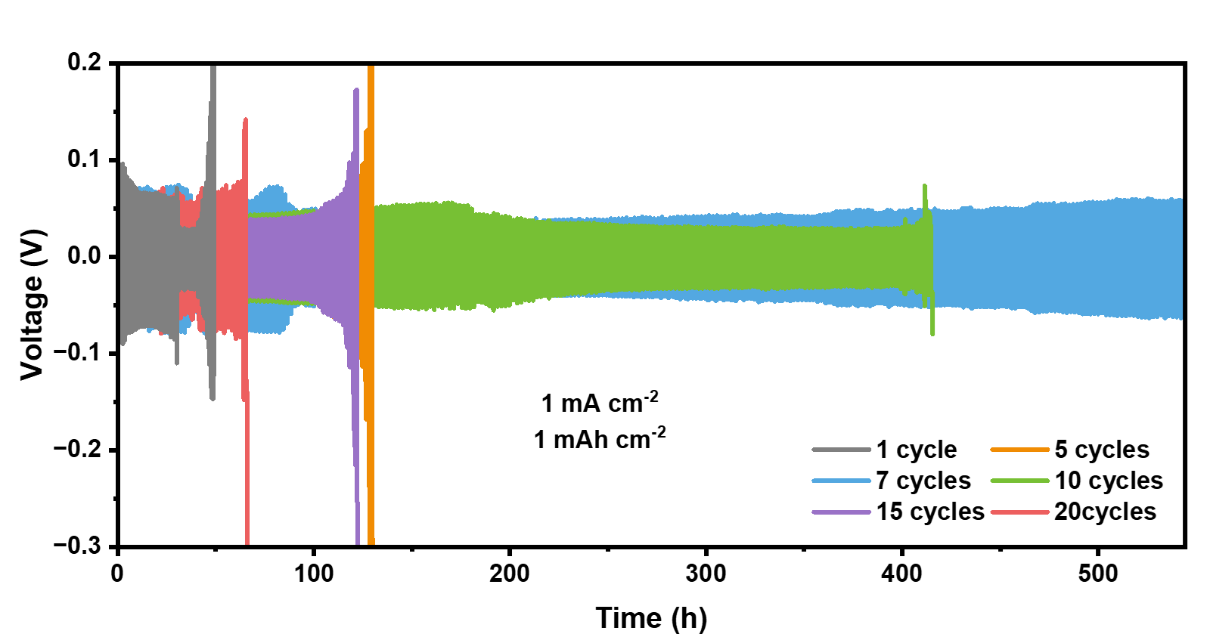


**Fig. S9** Long-term galvanostatic cycling of symmetric cells in aqueous electrolytes with electrodes prepared by OAPC process with electrolyte (PC: water = 3:2) through different cycles

The Zn electrodes were pre-treated in the symmetric cells by discharging and charging for 1, 5, 7, 10, 15 or 20 cycles. As shown in Fig. S9, the Zn electrodes obtained within 7 cycles displays the best stability among all the candidates. Therefore, we conducted the OAPC process for 7 cycles for follow-up experiments and tests.


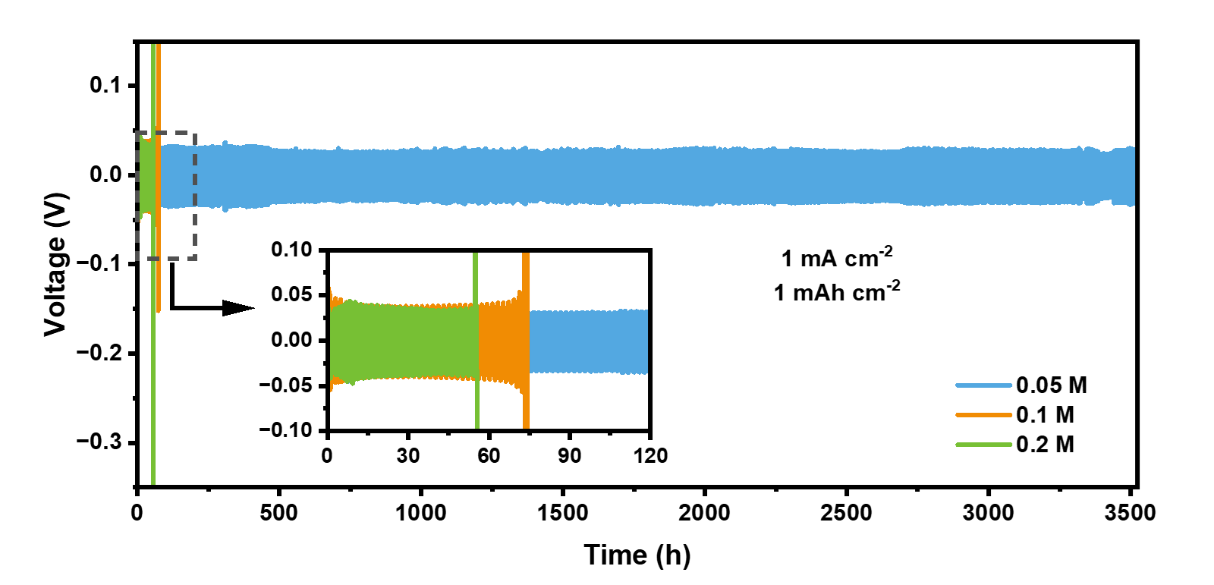


**Fig. S10** Long-term galvanostatic cycling of symmetric cells in aqueous electrolytes with electrodes prepared by OAPC process with electrolyte (PC: water = 3:2) containing different IPS concentrations through 7 cycles.

The organic electrolytes for OAPC process were prepared by adding 0.05 M, 0.1 M or 0.2 M IPS to electrolyte (PC: water = 3:2). Then, symmetrical cells with the Zn anodes from the electrolytes with 0.05 M IPS shows the best result, which is named OIL-IPS@Zn.

**
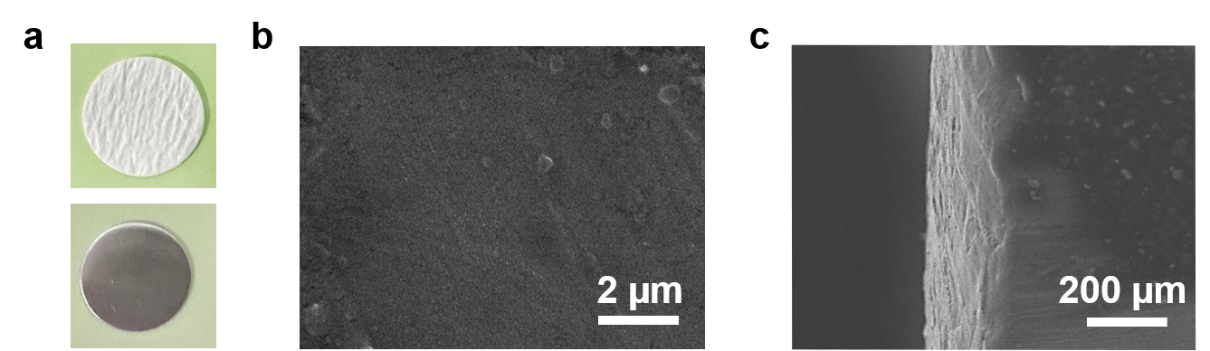
**

**Fig. S11** (**a**) Digital images of Glass microfiber filter (GF/F) and zinc foil before pretreatment. (**b**) SEM image, and (**c**) cross-section SEM image of zinc foil before pretreatment

**
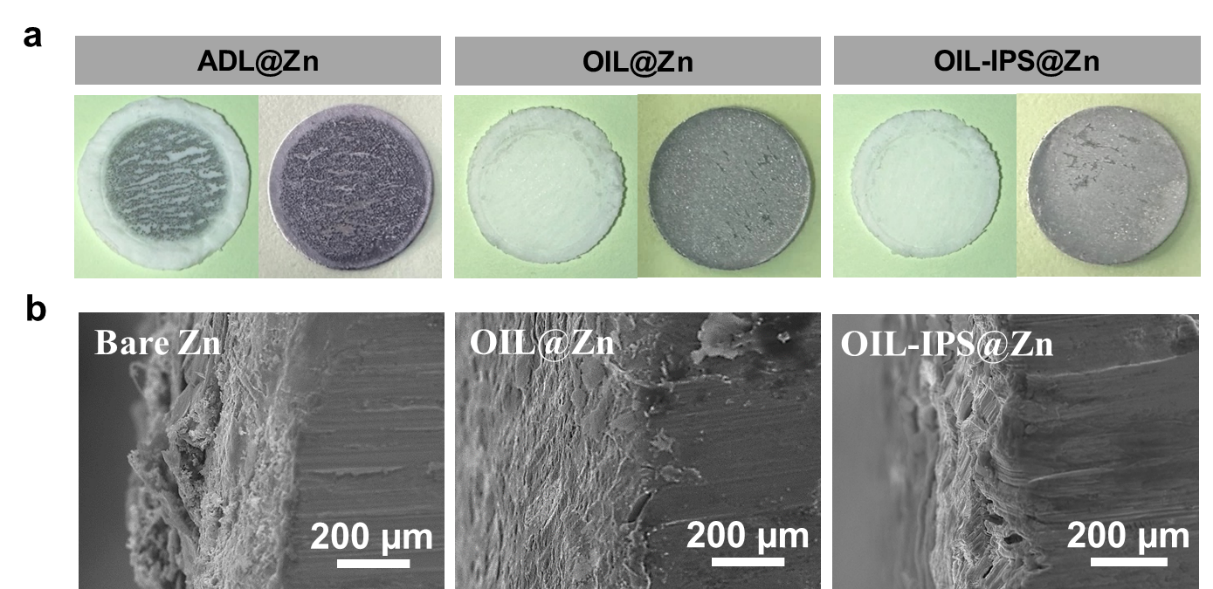
**

**Fig. S12** (**a**) Digital images of glass microfiber filters (GF/F) and electrodes, and (**b**) cross-section SEM images for ADL@Zn, OIL@Zn and OIL-IPS@Zn

**
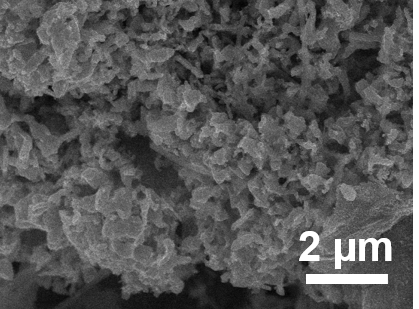
**

**Fig. S13** SEM image of bare Zn after cycling in the aqueous electrolyte with the addition of IPS

Compared with the uniform morphology of OIL-IPS@Zn, the dendrite growth of bare Zn surface after cycling in the aqueous electrolyte with the addition of IPS is very obvious, which indicates that IPS as an additive of aqueous electrolyte can not significantly improve the dendrite growth and side reactions of zinc anode, while the effect of the OAPC process is very significant.


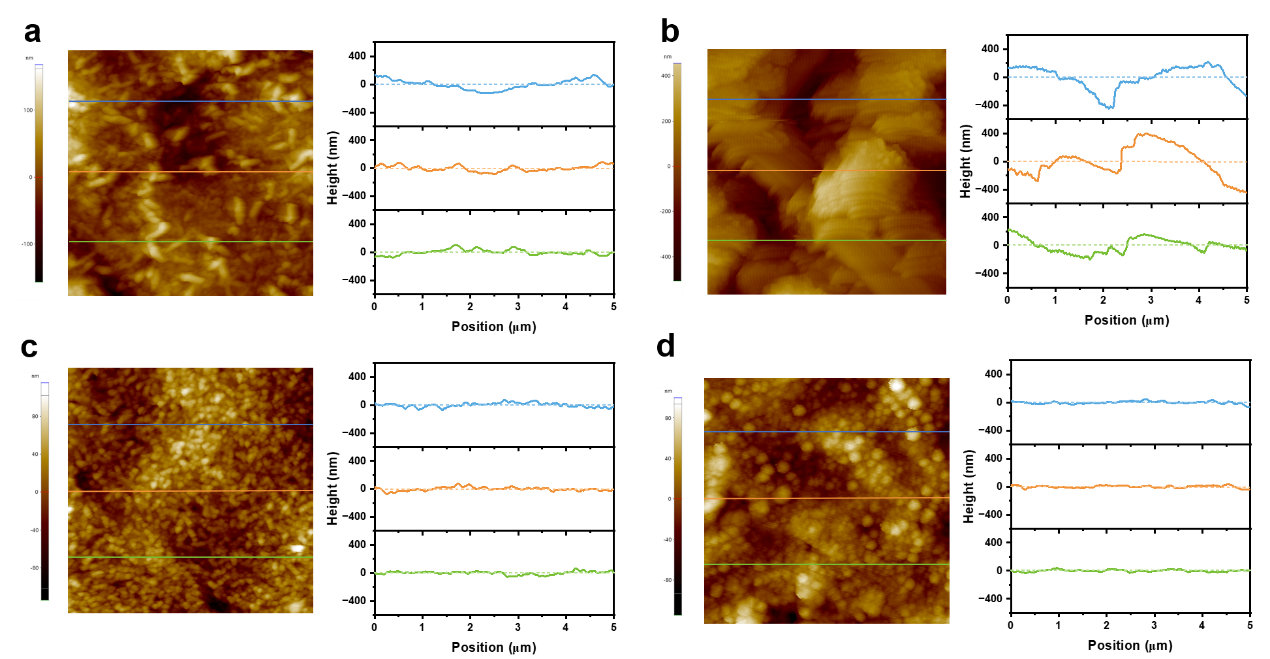


**Fig. S14** AFM images and line height distribution of (**a**) Bare Zn, (**b**) ADL@Zn, (**c**) OIL@Zn and (**d**) OIL-IPS@Zn electrodes


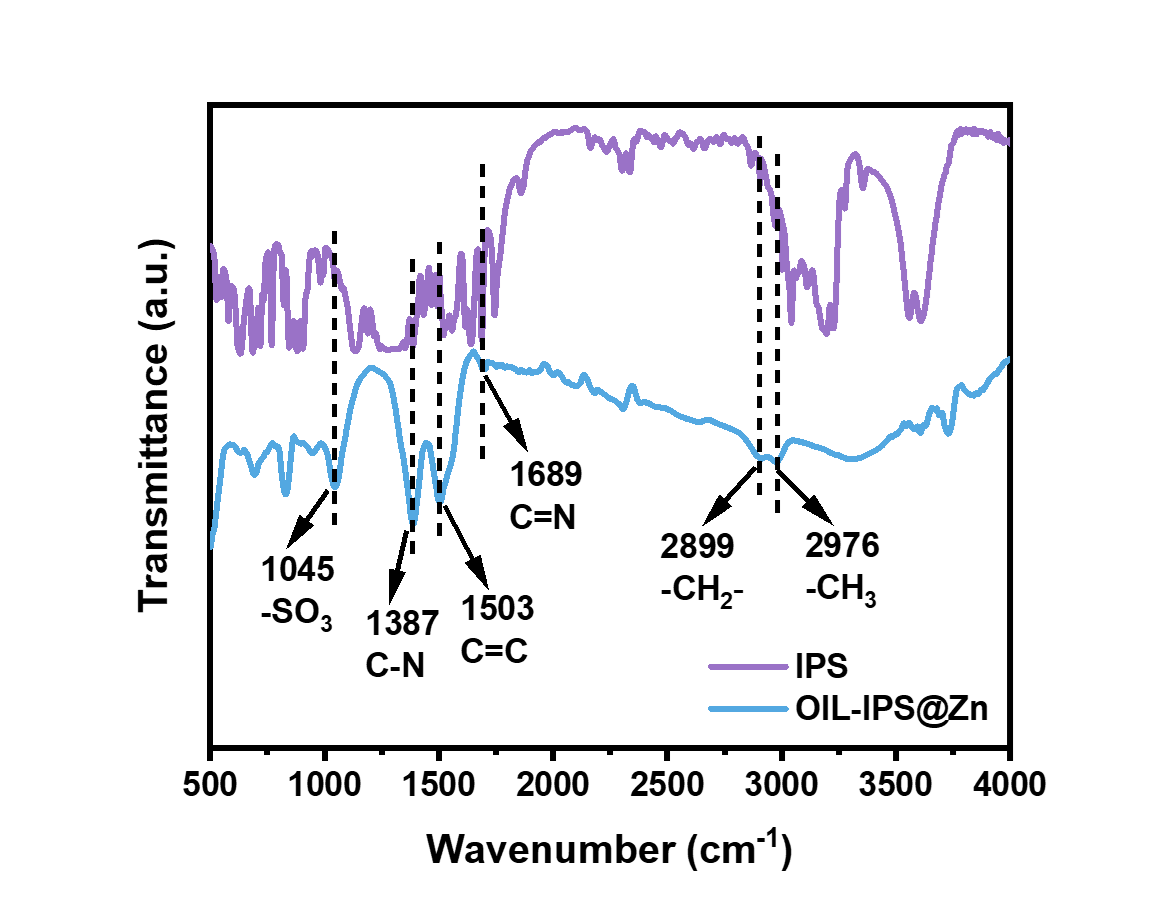


**Fig. S15** FTIR of OIL-IPS@Zn and IPS

The characteristic peaks of IPS can be found in OIL-IPS@Zn, indicating the successful insertion of IPS in OIL.


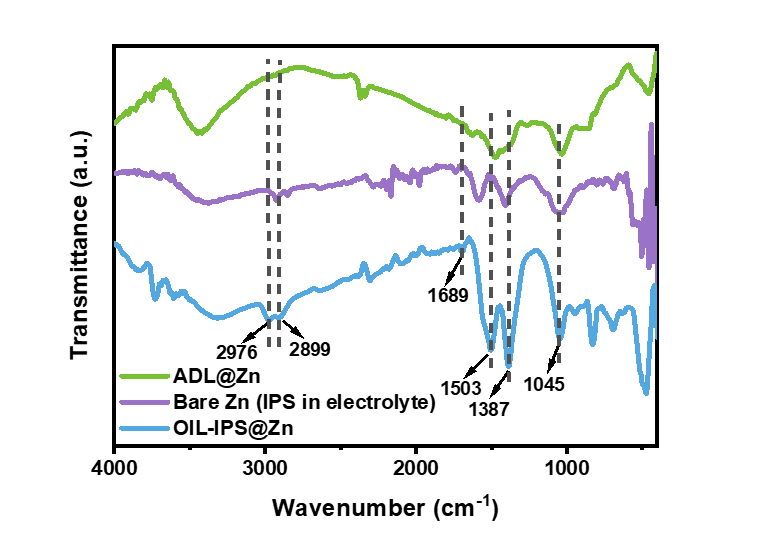


**Fig. S16** FTIR spectrum of ADL@Zn, OIL-IPS@Zn and bare Zn after cycling in the aqueous electrolyte with the addition of IPS

FTIR results indicate that IPS molecules are only present in OIL-IPS@Zn, which indicates that IPS can only be successfully inserted into OIL during OAPC process, consistent with the EDS mapping results.


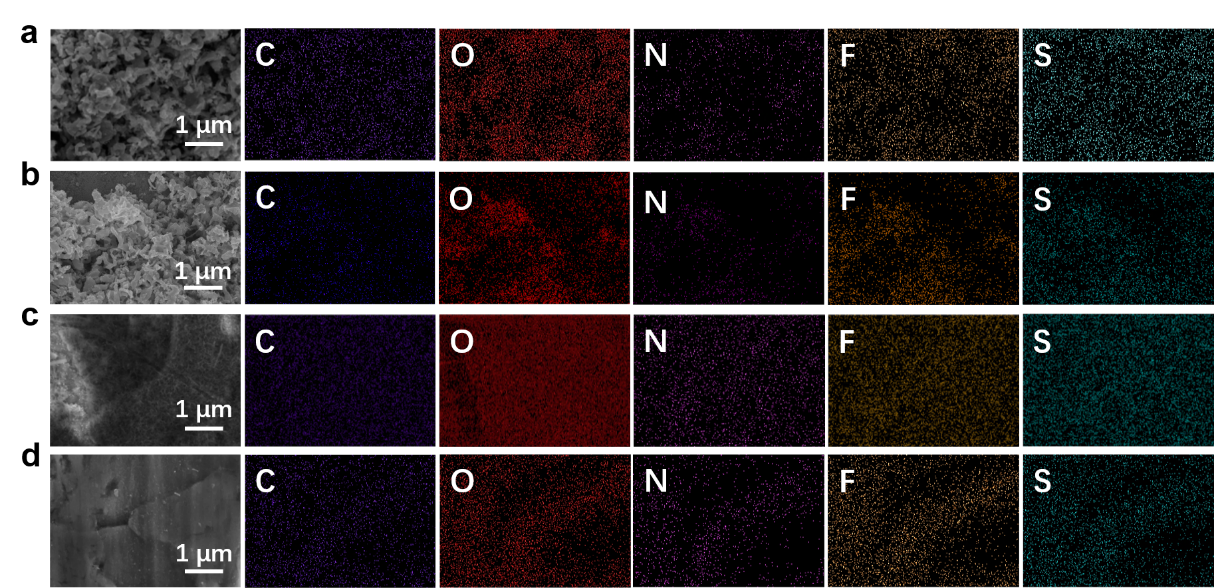


**Fig. S17** EDS mapping of (**a**) ADL@Zn, (**b**) bare Zn after cycling in the aqueous electrolyte with the addition of IPS, (**c**) OIL@Zn, (**d**) OIL-IPS@Zn. The actual element ratios are in Table S2

The EDS mapping results of the electrodes indicate the uniform distribution of C, O and N on these pre-treated Zn electrodes. Both electrodes from aqueous electrodes (ADL@Zn and bare Zn after cycling in the aqueous electrolyte with the addition of IPS) show minimum S and low F distribution, indicating that the SEI layers are dominated by inorganic compounds (ZnF_2_, ZnO, and Zn(OH)_2_, *etc.*) On the other hand, the electrodes from organic electrolytes show much higher S and F content, relating to the increase in organic content in OIL from the insertion of ions and decomposition of PC molecules. Furthermore, OIL-IPS@Zn shows the existence of N in the surface layer, referring to the IPS insertion in the OIL. Interestingly, the content of N in bare Zn after cycling in the aqueous electrolyte with the addition of IPS is zero, which indicates that the water-derived SEI layer cannot fix IPS while the organic-derived ones can easily retain the organic structures.


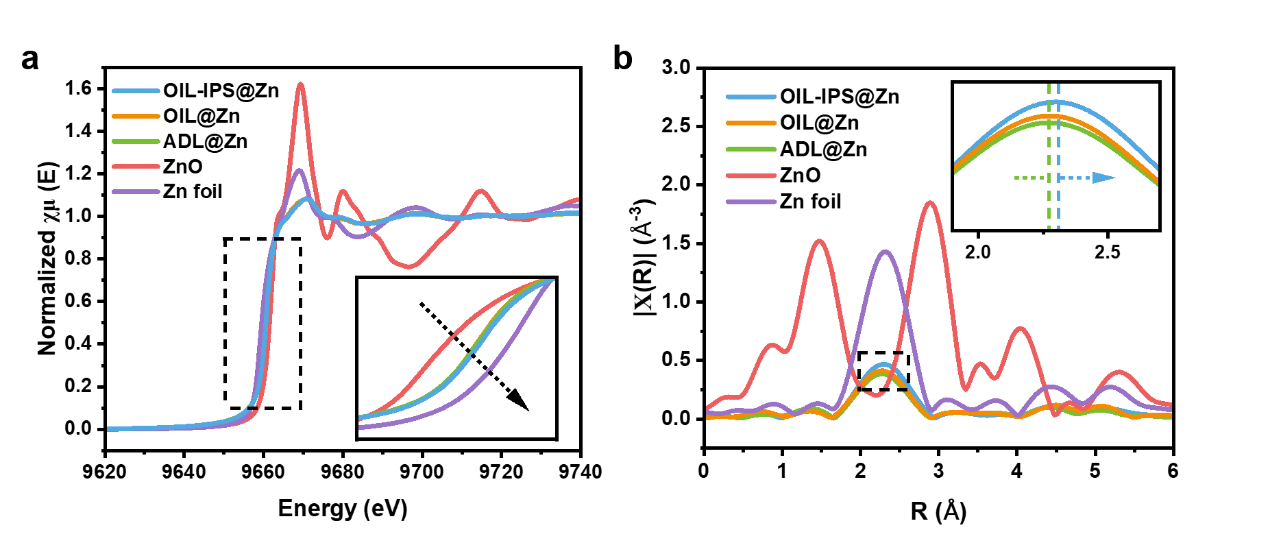
 **Fig. S18** (**a**) Zn K-edge XANES and (**b**) Zn K-edge EXAFS spectra of OIL-IPS@Zn, OIL@Zn, ADL@Zn, ZnO and Zn foil

All the pre-treated surfaces of Zn anodes show oxidation states between 0 and +2, referring to the surface composition of metallic and Zn^2+^ in the surface layers. However, the Zn-Zn peak in OIL-IPS@Zn shows a slight shift compared to the other candidates, indicating that there are additional interactions between Zn and the inserted IPS molecules. This result is consistent with the DFT calculation prediction in Fig. 1b, which also verifies the success incorporation if IPS molecules in the functional OIL.


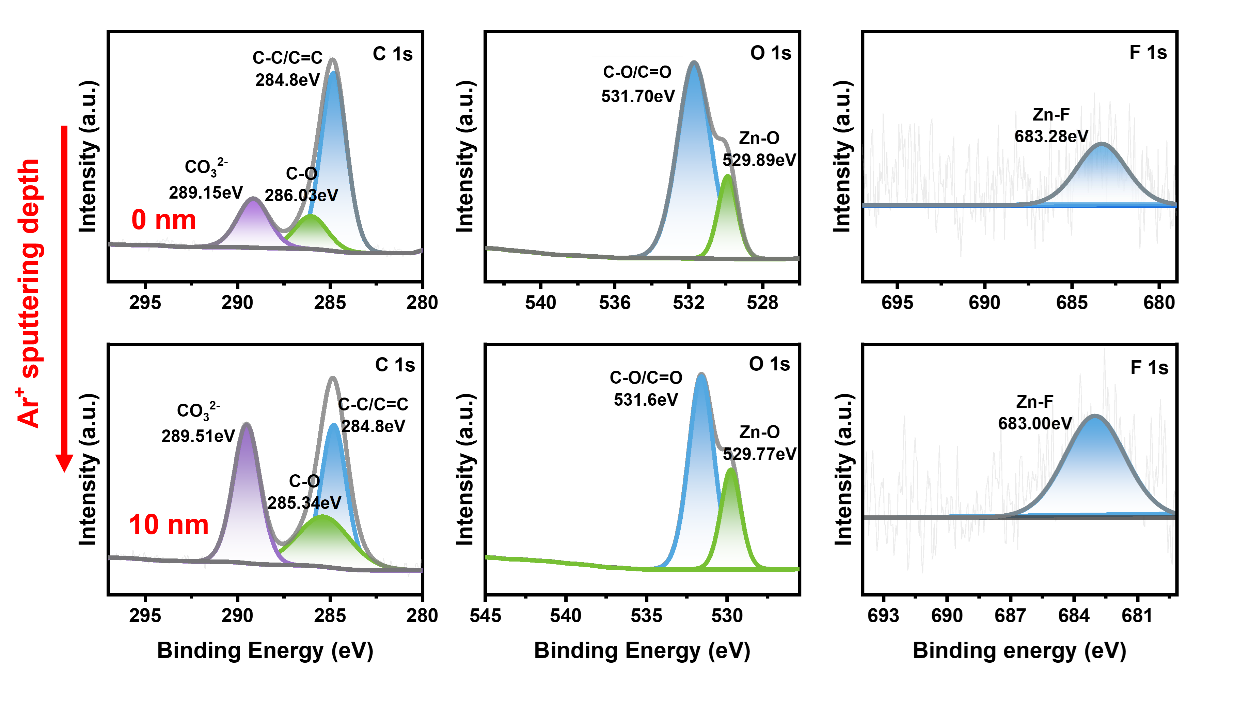


**Fig. S19** ADL@Zn anode spectra of C 1s, O 1s and F 1s XPS at different depths

The element composition at different depths in the ADL@Zn shows the same results, indicating the SEI layers are homogeneously distributed and no variation can be found in the chemical compositions.


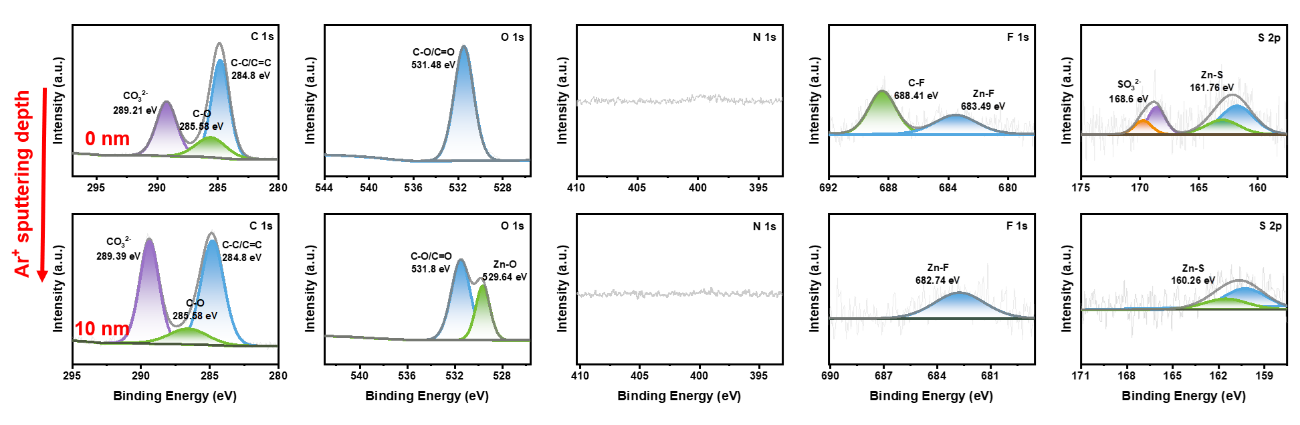


**Fig. S20** OIL@Zn anode spectra of C 1s, O 1s, N 1s, F 1s and S 2p XPS at different depths

No N element is found in OIL@Zn, indicating the N element found in OIL-IPS originates from IPS.


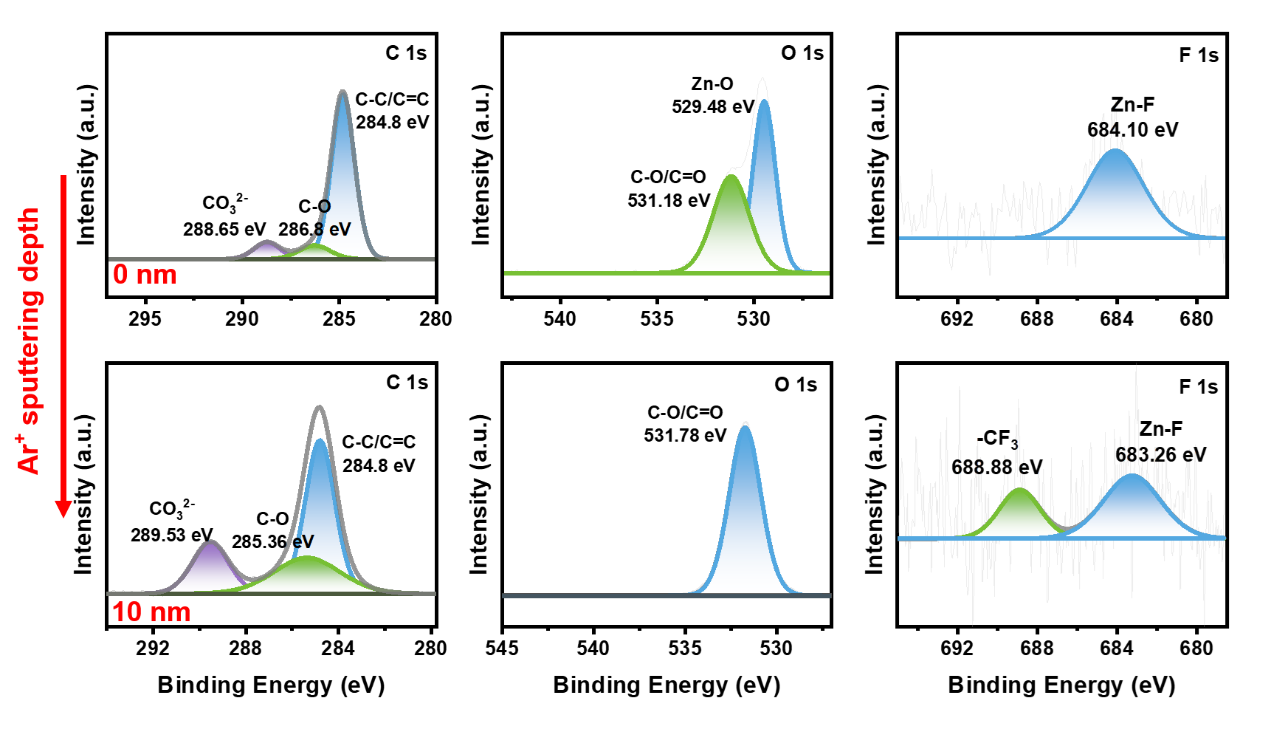


**Fig. S21** OIL-IPS@Zn anode spectra of C 1s, O 1s and F 1s XPS at different depths

**
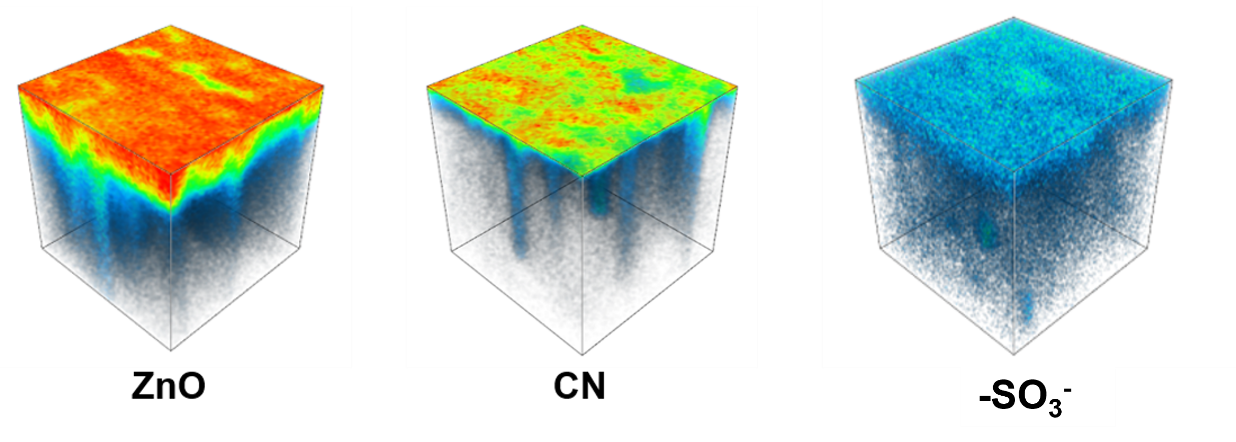
**

**Fig. S22** TOF-SIMS analysis of OIL-IPS@Zn

The result shows a similar trend to the XPS in that the N related to the imidazolium group is mainly distributed on the surface, and -SO_3_^-^ is distributed on the bottom of SEI.

**
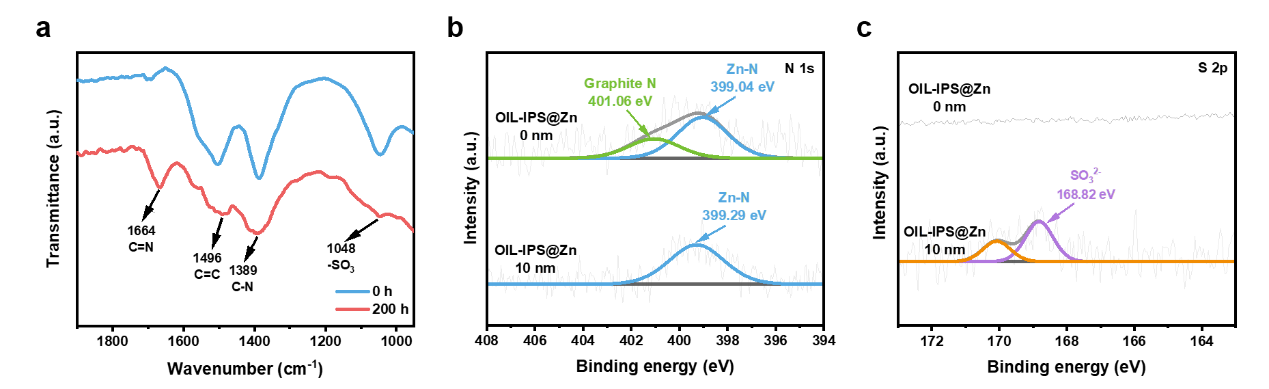
**

**Fig. S23** (**a**) FTIR spectrum of OIL-IPS@Zn anode after long-term cycling. OIL-IPS@Zn anode (after 200 h) spectra of (**b**) N 1s, and (**c**) S 2p XPS at different depths

**
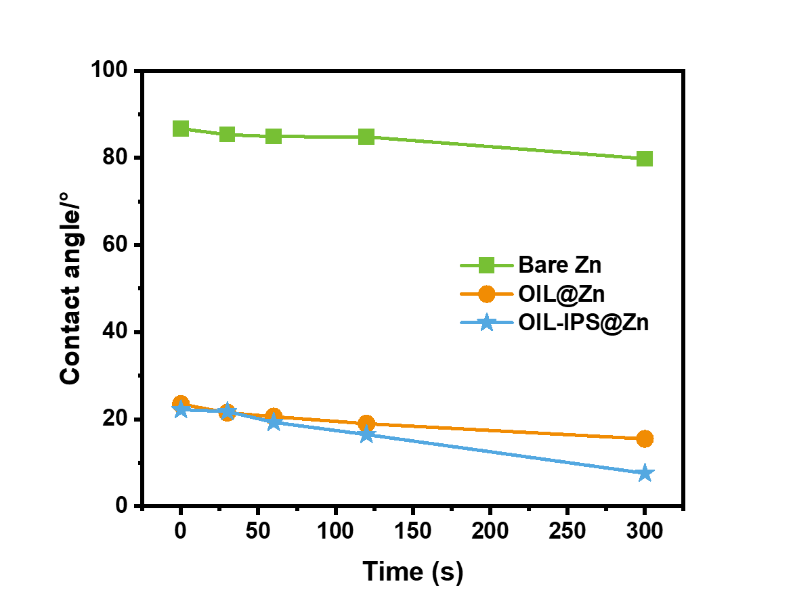
**

**Fig. S24** Comparison of contact angles variation trend of the aqueous electrolyte on the electrodes

**
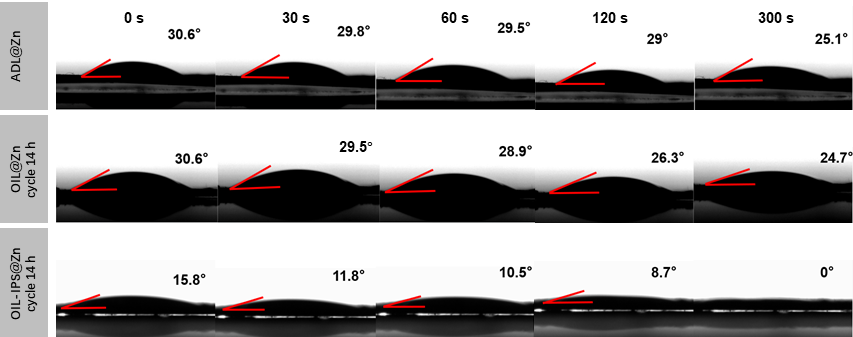
**

**Fig. S25** Contact angles of the aqueous electrolyte on the electrodes after cycles


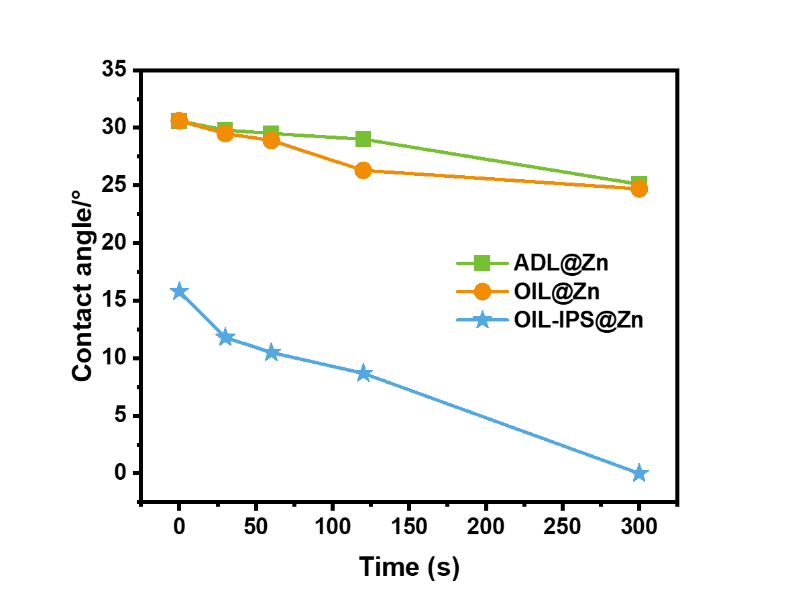


**Fig. S26** Comparison of contact angles variation trend of the aqueous electrolyte on the electrodes after cycles


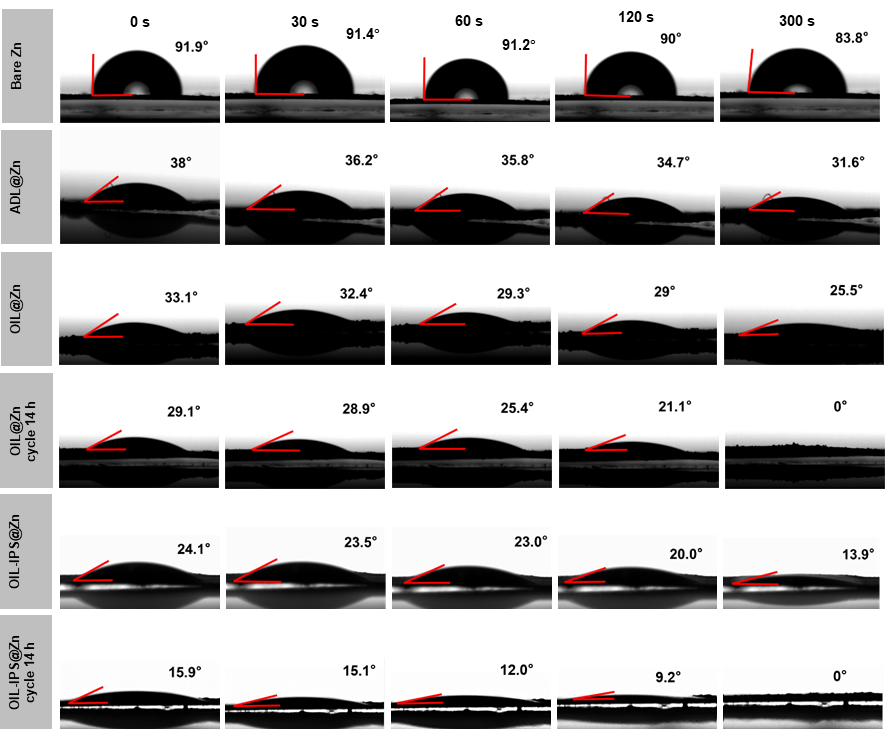


**Fig. S27** Contact angles of water on the electrodes (before and after cycles)

Both electrodes from the OAPC process display excellent affinity towards water, even after cycling.


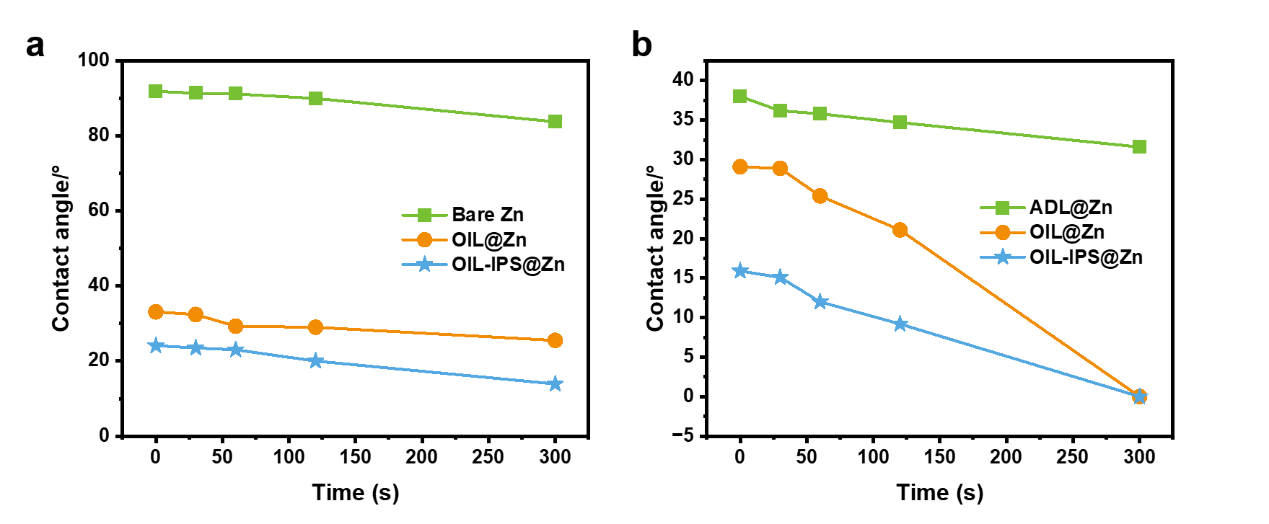


**Fig. S28** Comparison of contact angles variation trend of water on the electrodes (**a**) before and (**b**) after cycles


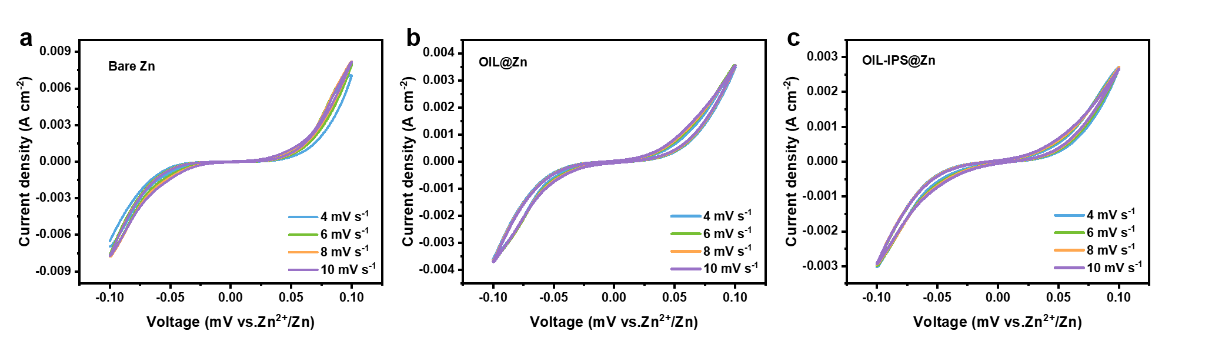


**Fig. S29** (**a**) Bare Zn, (**b**) OIL@Zn, (**c**) OIL-IPS@Zn CV curve of a symmetrical battery


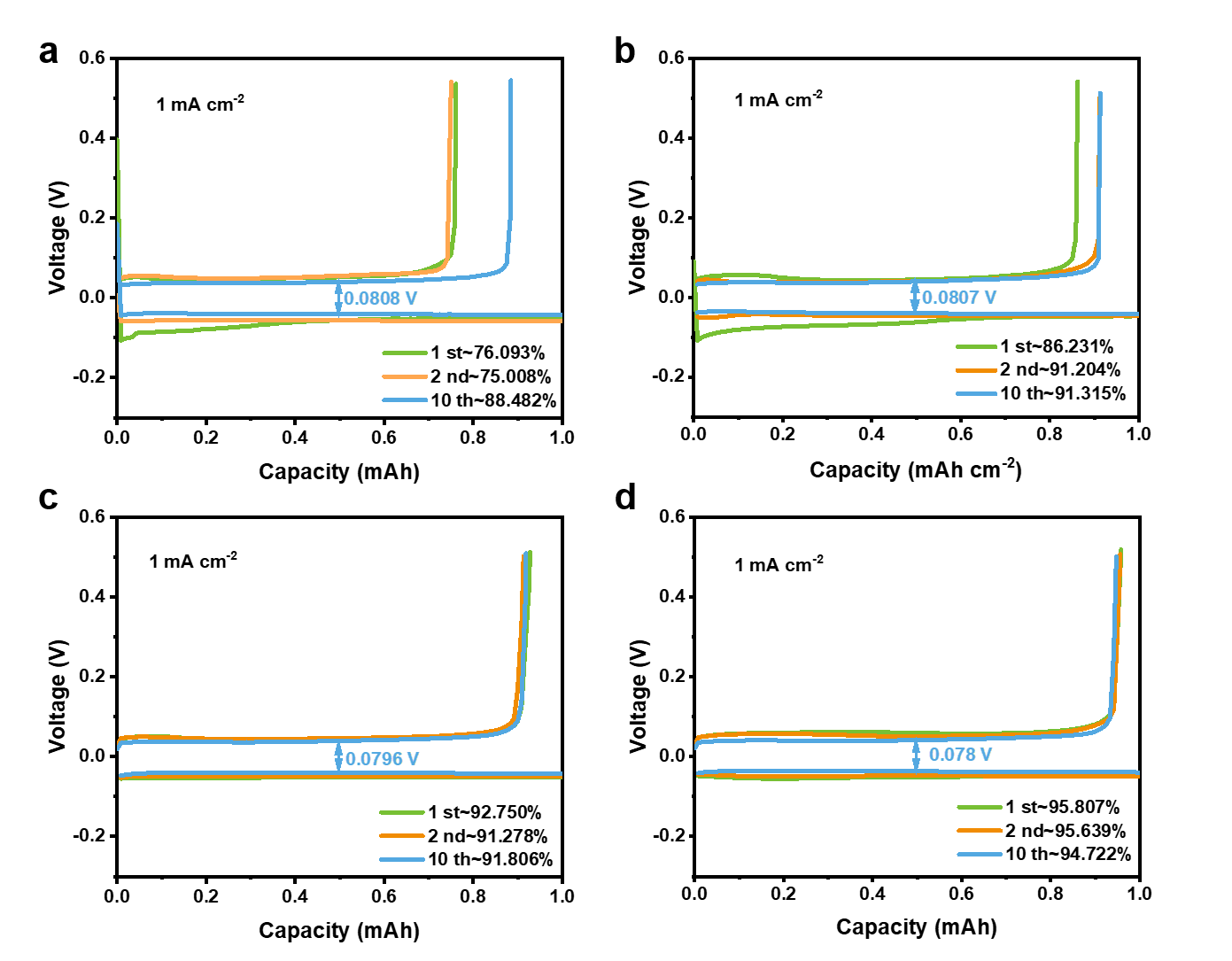


**Fig. S30** Charge and discharge curves of the first, second and tenth cycles of (**a**) Bare Zn||Cu, (**b**) Bare Zn||Cu with IPS addition in the electrolyte, (**c**) OIL@Zn||Cu, and (**d**) OIL-IPS@Zn||Cu

The OIL-IPS shows the lowest overpotential, indicating the functional OIL benefit the charge transfer and facilitate the smooth Zn plating/stripping.


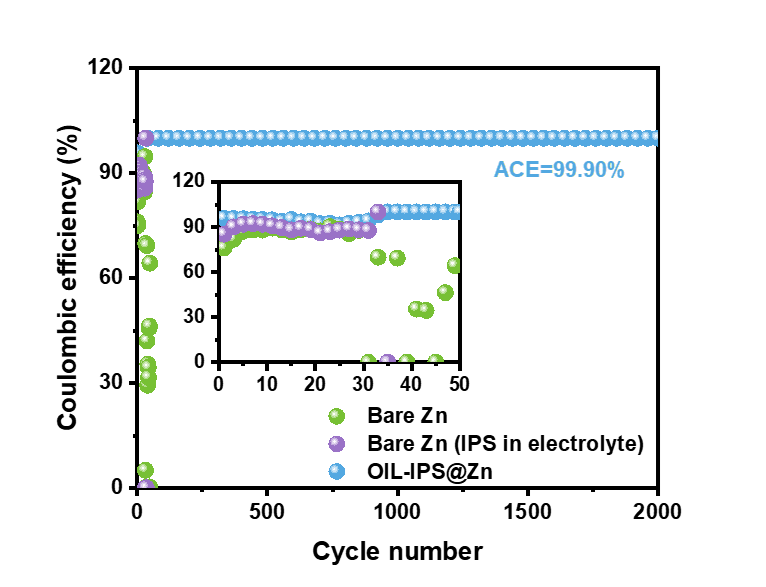


**Fig. S31** Comparison of CE between Bare Zn||Cu, Bare Zn||Cu with IPS addition in the electrolyte and OIL-IPS@Zn||Cu half cells during cycles at 1 mA cm^-2^ with an areal capacity of 1 mAh cm^-2^


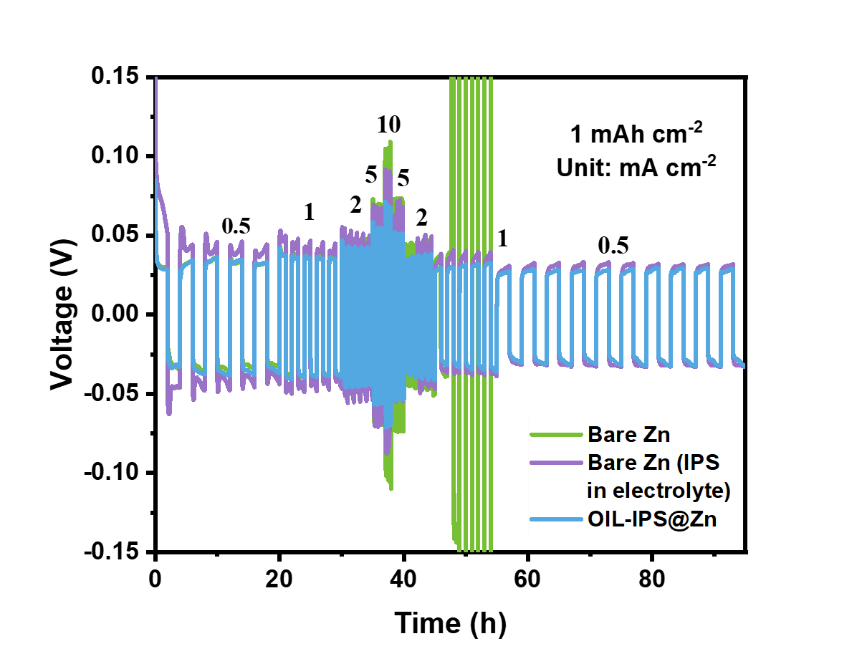


**Fig. S32** Rate capability of symmetrical cells assembled using Bare Zn, Bare Zn with IPS addition in the electrolyte and OIL-IPS@Zn anodes at various current densities from 0.5 to 10 mA cm^-2^

The results indicate that OIL-IPS@Zn from the OAPC process has a better rate capability than the bare Zn with IPS added to the electrolyte for cycling, indicating that IPS brush layer can only be well preserved in the organic-derived SEI layer.


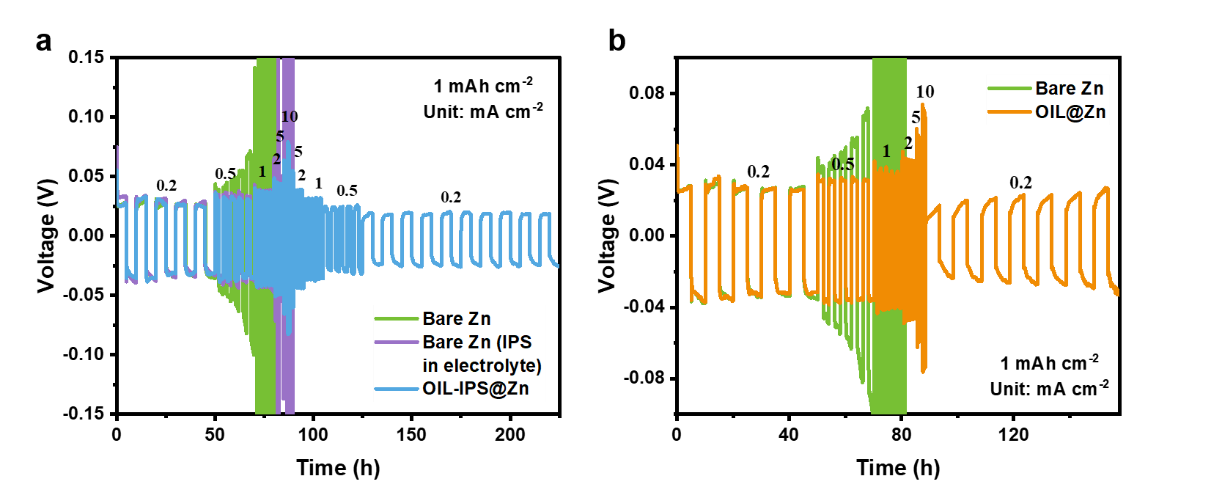


**Fig. S33** Rate capability of symmetrical cells assembled using (**a**) Bare Zn, Bare Zn with IPS addition in the electrolyte and OIL-IPS@Zn, (**b**) Bare Zn and OIL@Zn at various current densities from 0.2 to 10 mA cm^-2^

**
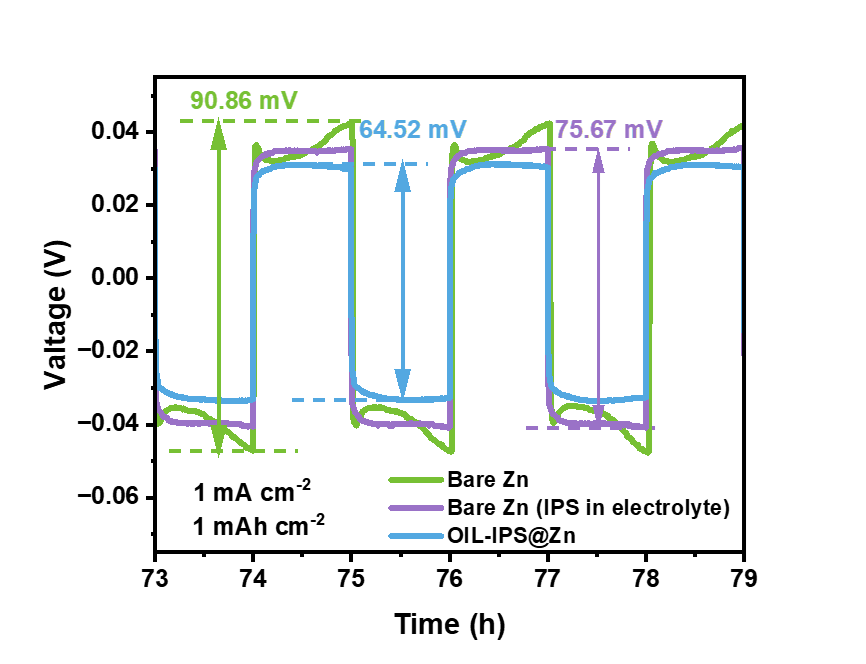
**

**Fig. S34** Voltage lag of Bare Zn, Bare Zn with IPS in the electrolyte and OIL-IPS@Zn symmetrical batteries

**
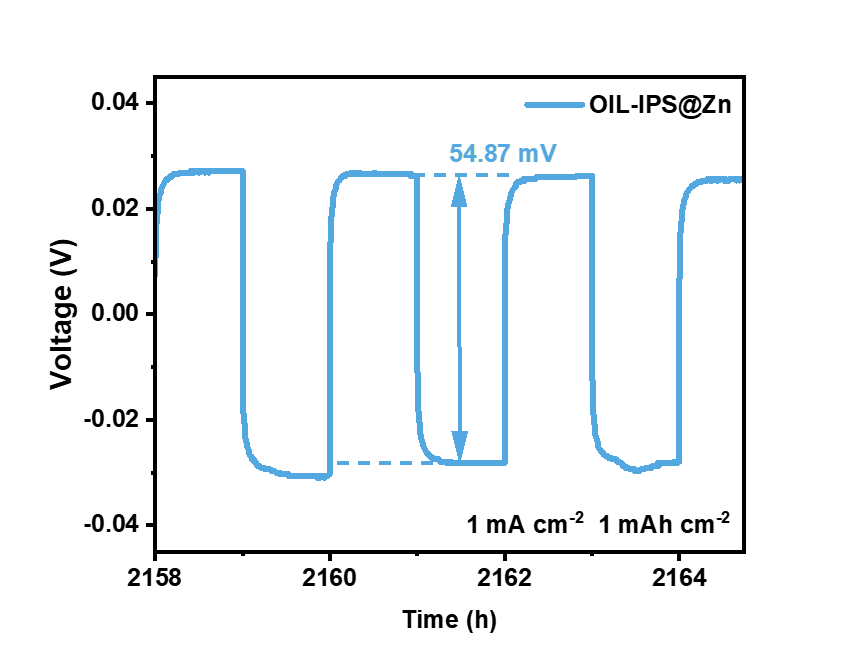
**

**Fig. S35** Voltage lag of OIL-IPS@Zn symmetrical battery


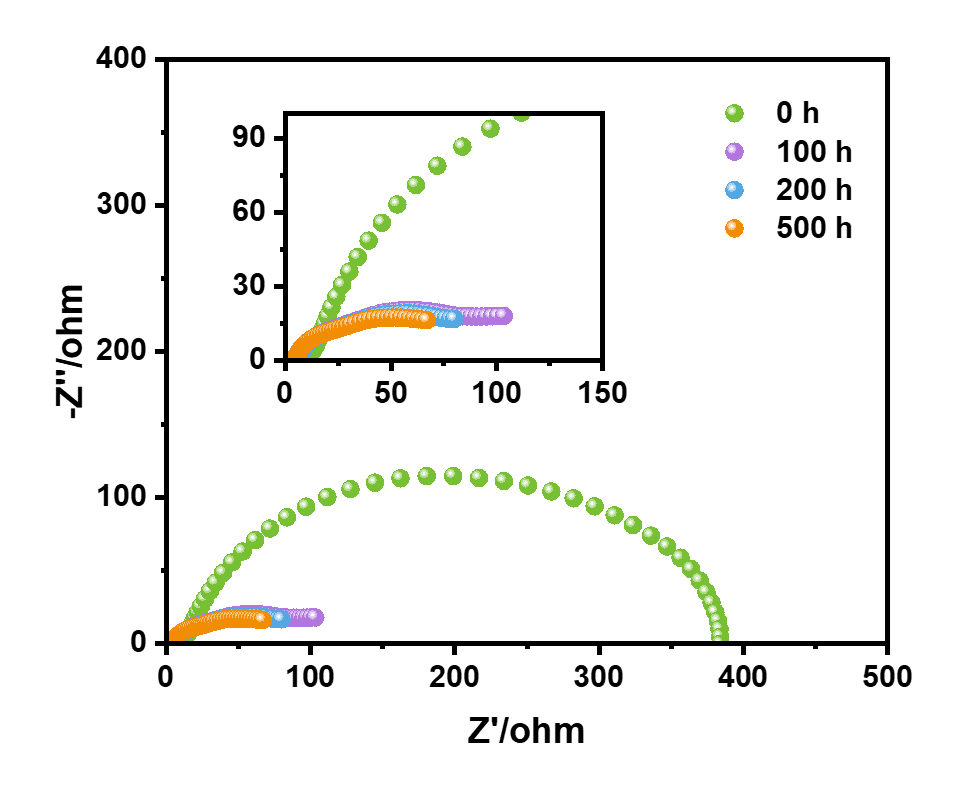


**Fig. S36** EIS images of OIL-IPS@Zn symmetric batteries before and after long-term cycling


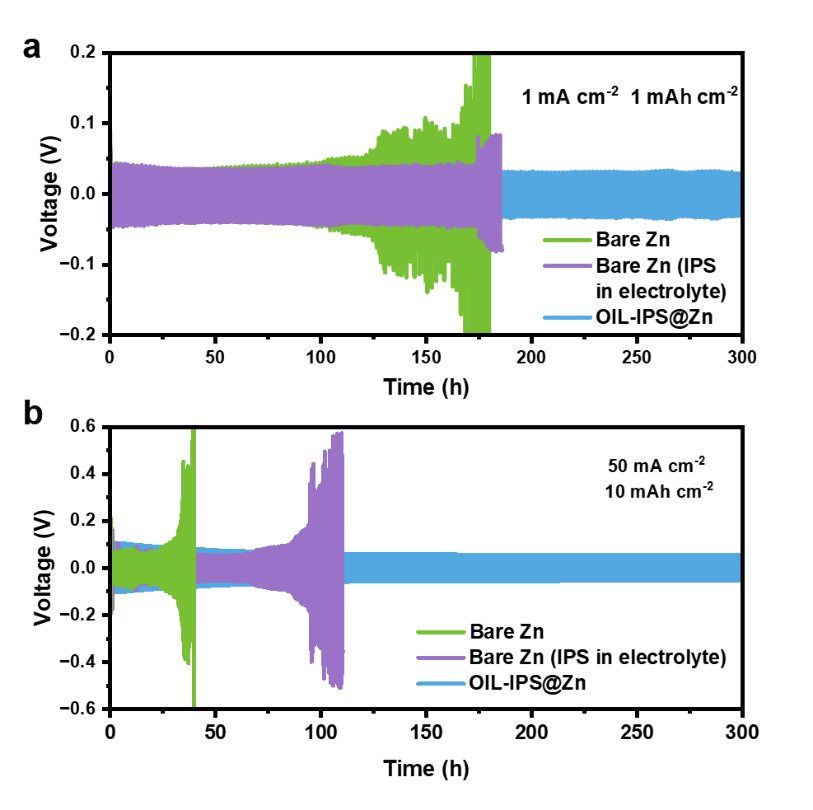


**Fig. S37** Long-term galvanostatic cycling of symmetric Zn cells with Bare Zn, Bare Zn with IPS addition in the electrolyte and OIL-IPS@Zn electrode at (**a**) 1 mA cm^−2^/1 mAh cm^−2^, and (**b**) 50 mA cm^−2^/10 mAh cm^−2^

The results indicate that OIL-IPS@Zn from the OAPC process has a better cycling capability than the bare Zn with IPS added to the electrolyte for cycling, indicating that IPS brush layer can only be well preserved in the organic-derived SEI layer. The direct introduction of IPS as an additive into aqueous electrolytes leads to the formation of an unstable SEI on the electrode surface. Due to the absence of organic solvents, the resulting SEI layer lacks mechanical strength and cannot achieve the robust structural integrity observed in the OIL-IPS@Zn system. In contrast, the incorporation of IPS into the SEI layer of OIL-IPS@Zn significantly enhances ion transport kinetics. Consequently, the straightforward addition of IPS to the electrolyte without pretreatment processes yields inferior performance compared to the OIL-IPS@Zn configuration.


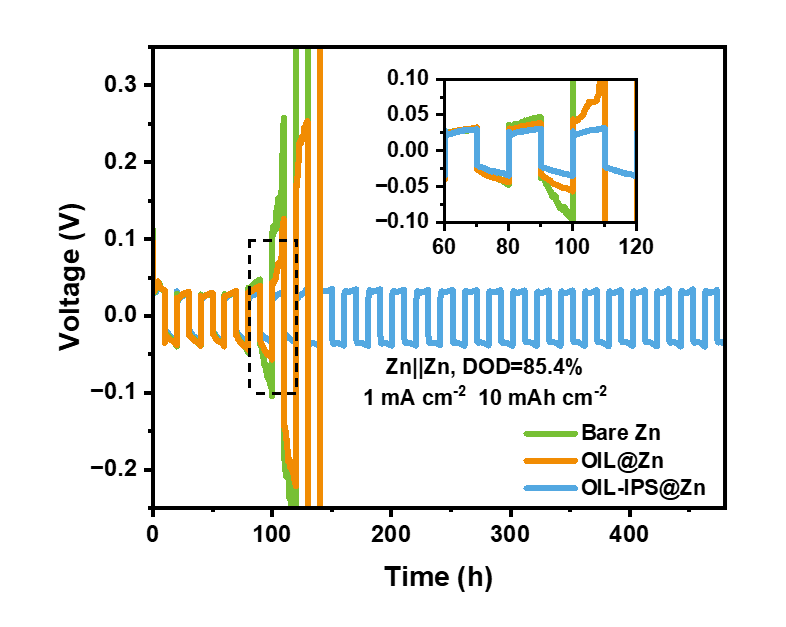


**Fig. S38** Long-term galvanostatic cycling of symmetric Zn cells of Bare Zn, OIL@Zn and OIL-IPS@Zn electrode with high DOD


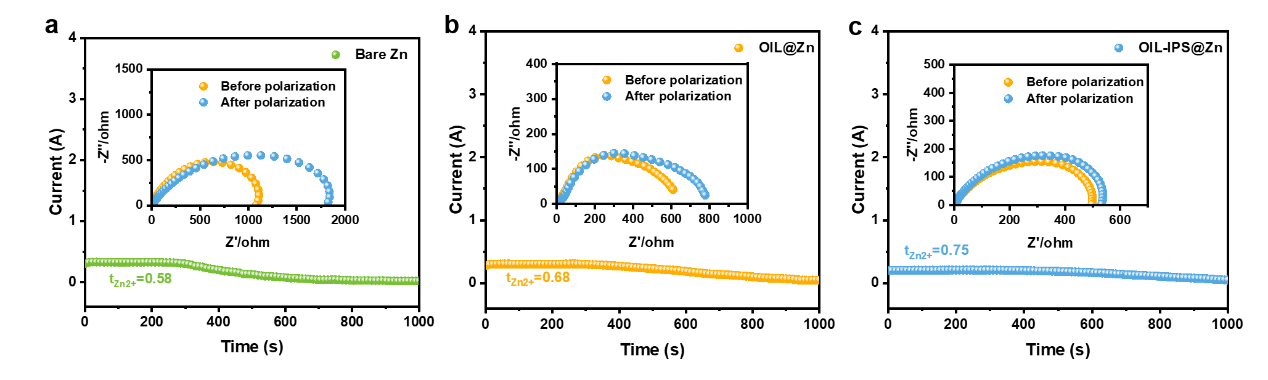


**Fig. S39** Zn^2+^ transference number of (**a**) Bare Zn, (**b**) OIL@Zn and (**c**) OIL-IPS@Zn


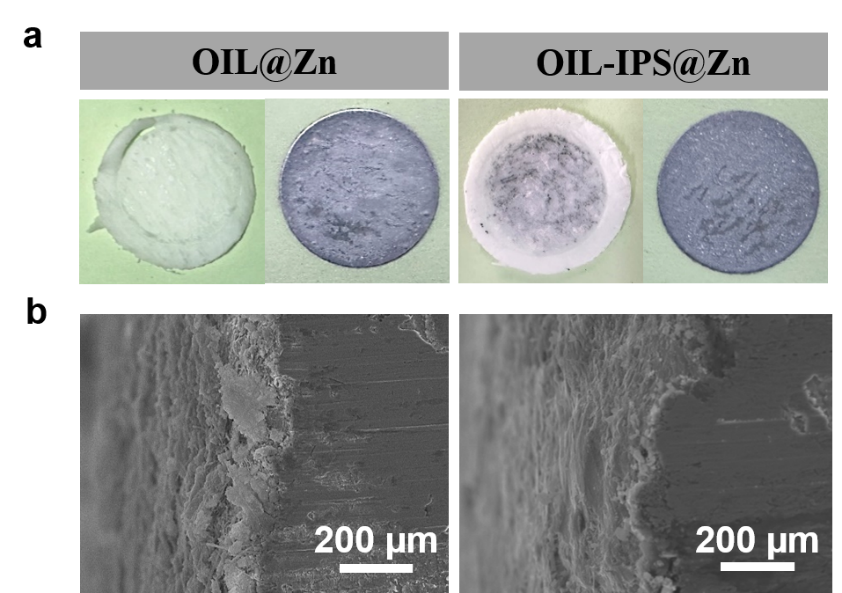


**Fig. S40** (**a**) Digital images of glass microfiber filters (GF/F) and electrodes after cycling for 10 cycles, and (**b**) cross-section SEM image for OIL@Zn and OIL-IPS@Zn after cycling


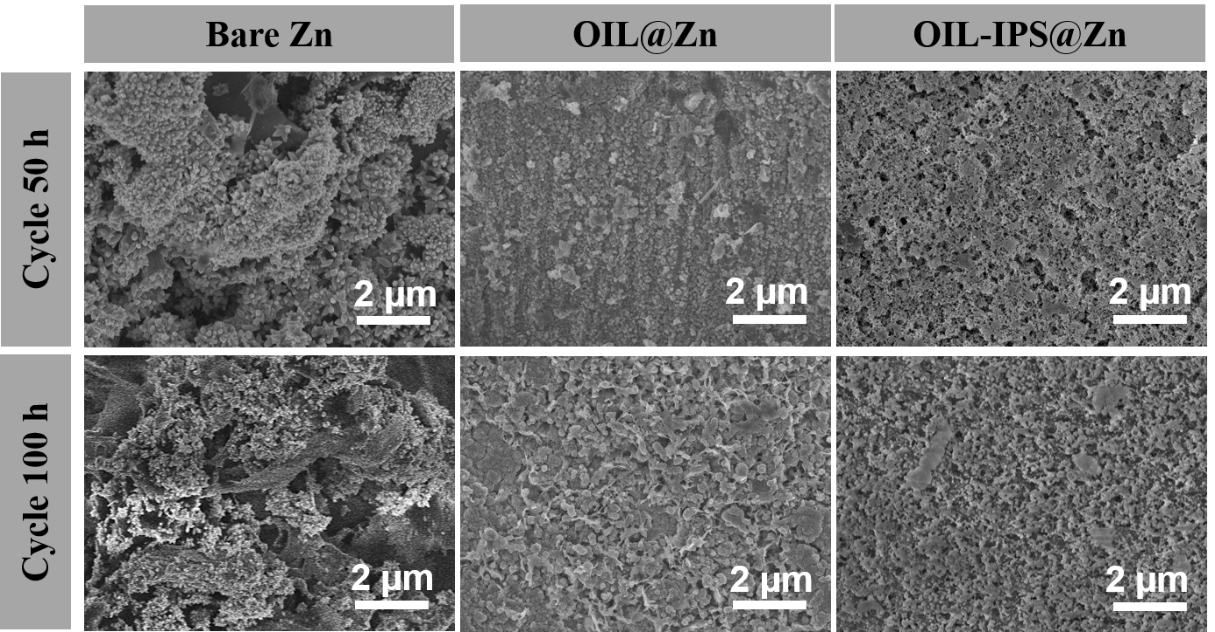


**Fig. S41** SEM images of Bare Zn, OIL@Zn and OIL-IPS@Zn after cycling in the aqueous electrolytes for 50 h and 100 h


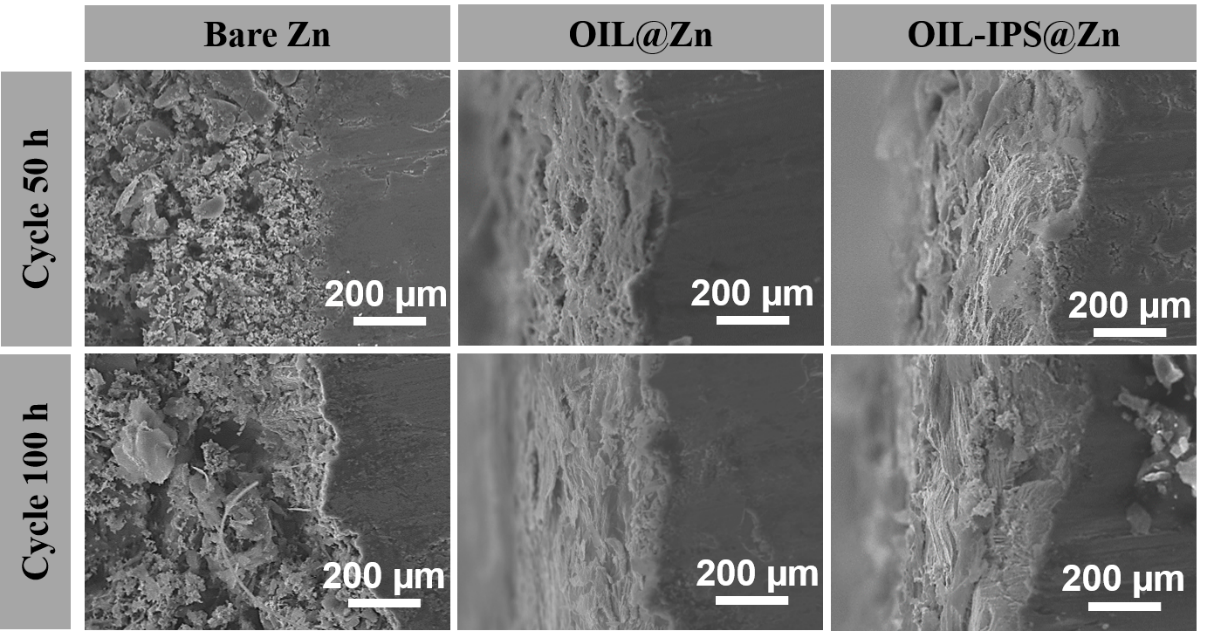


**Fig. S42** Cross-section SEM images of Bare Zn, OIL@Zn and OIL-IPS@Zn after cycling in the aqueous electrolyte for 50 h and 100 h


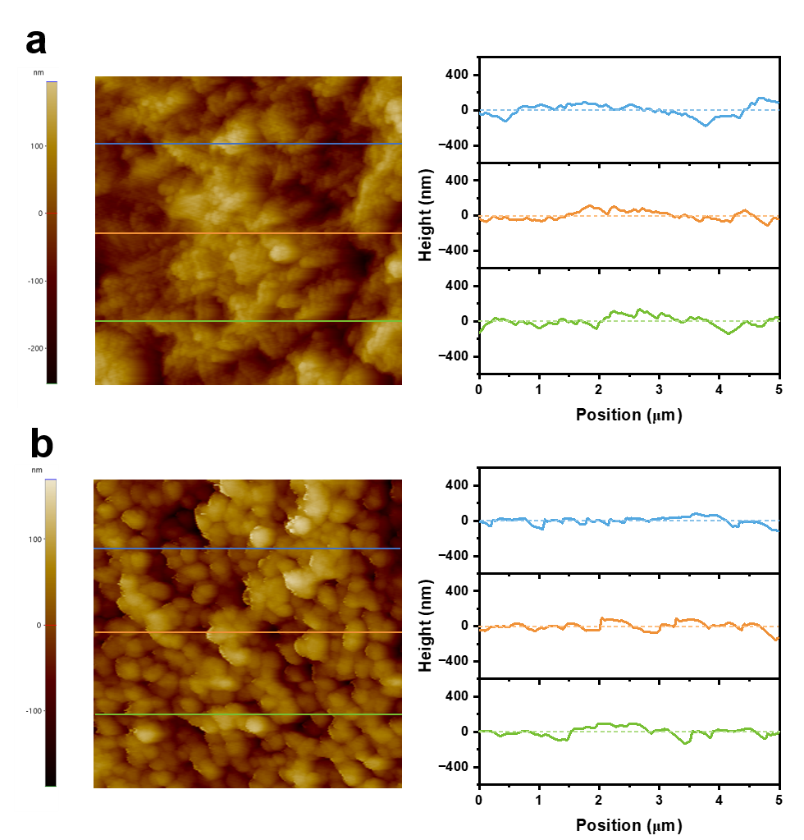


**Fig. S43** AFM images and line height distribution of OIL@Zn and OIL-IPS@Zn electrodes deposited at 1 mA cm^-2^,1 mAh cm^-2^ after 14 h


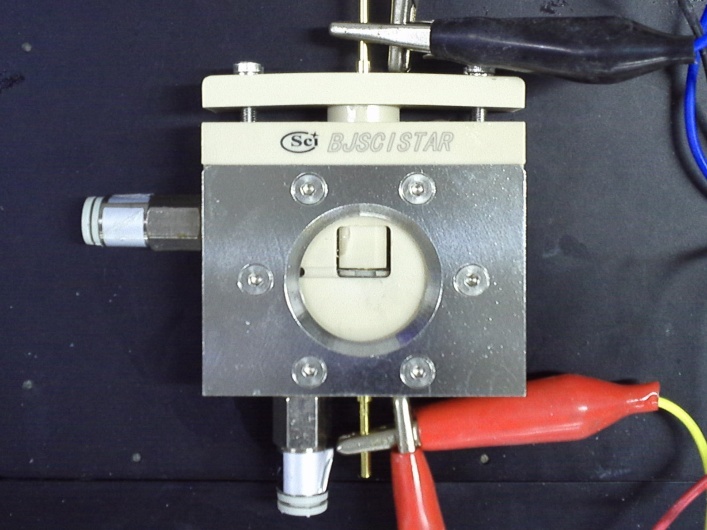


**Fig. S44** In-situ optical microscopy test sample pool


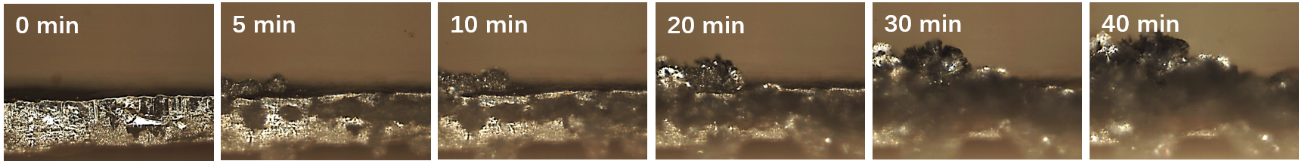


**Fig. S45** In-situ optical images of Bare Zn in IPS-added aqueous electrolytes at a current density of 5 mA cm^-2^

This result indicates that the IPS brush layer can only be well preserved in the organic-derived SEI layer which shows excellent capability in Zn plating/stripping and dendrite suppression.

**
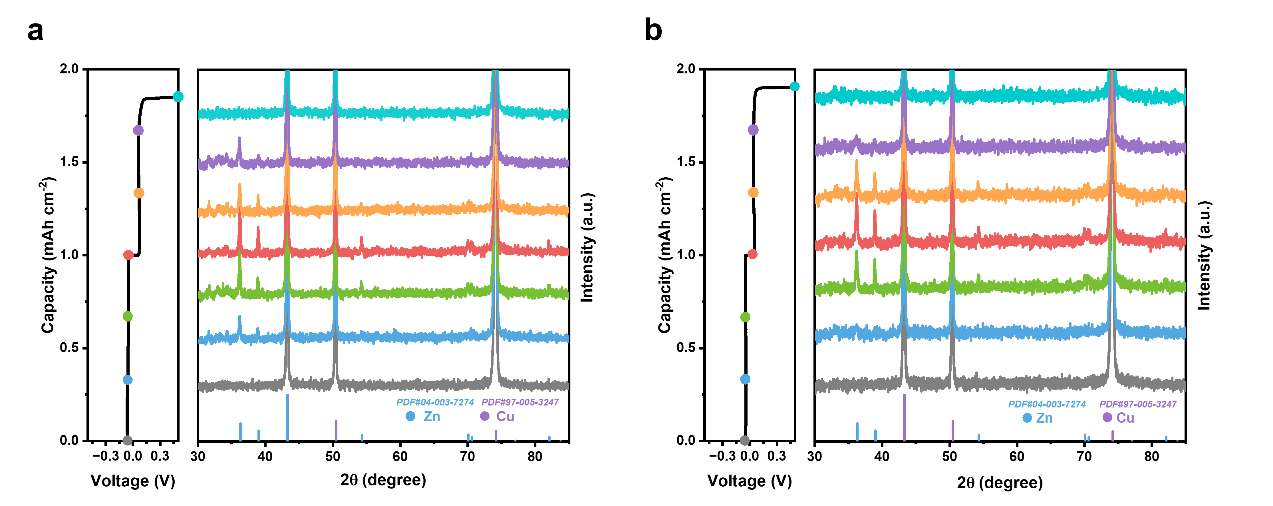
**

**Fig. S46** Ex-situ XRD pattern of the Cu electrode during the first Zn plating/stripping cycle of (**a**) Bare Zn||Cu, (**b**) OIL@Zn||Cu

**
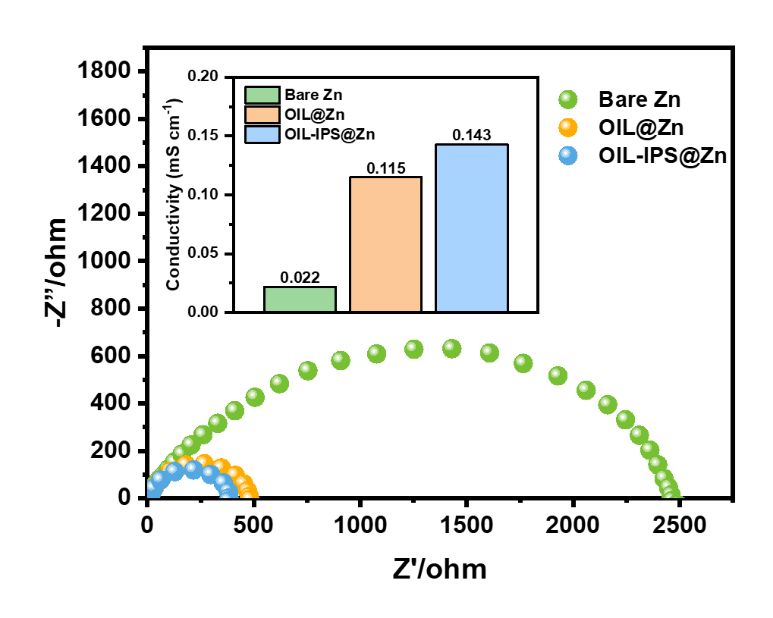
**

**Fig. S47** The ion conductivity of Bare Zn, OIL@Zn and OIL-IPS@Zn

**
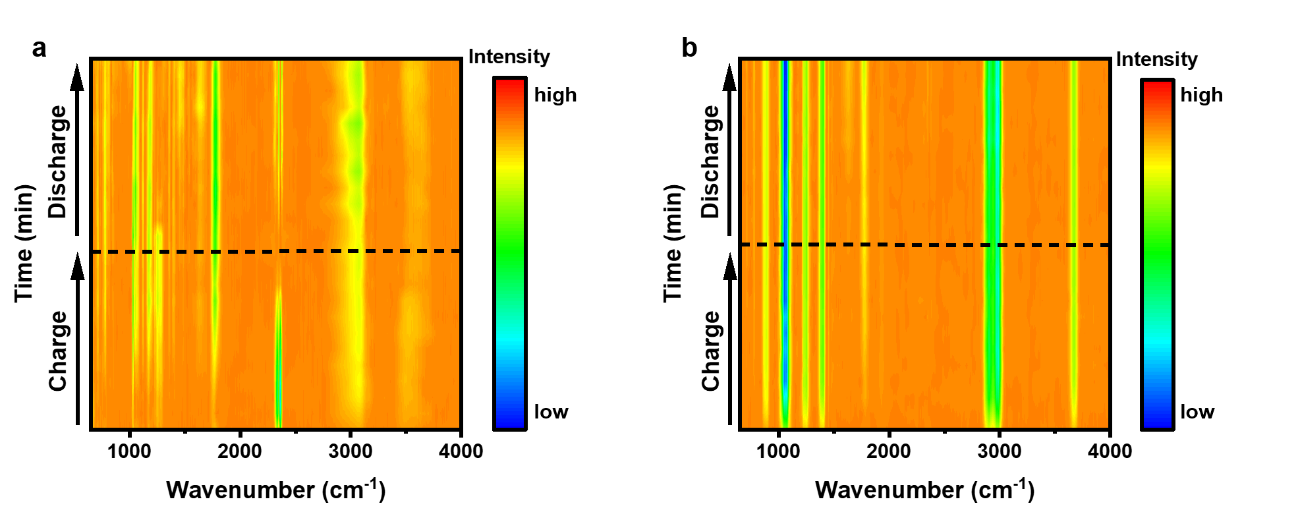
**

**Fig. S48** In-situ ATR-SEIRAS results in (**a**) mixture electrolyte (PC: water = 3:2), and (**b**) mixture electrolyte (PC: water = 3:2) with 0.05 M IPS of symmetrical cells


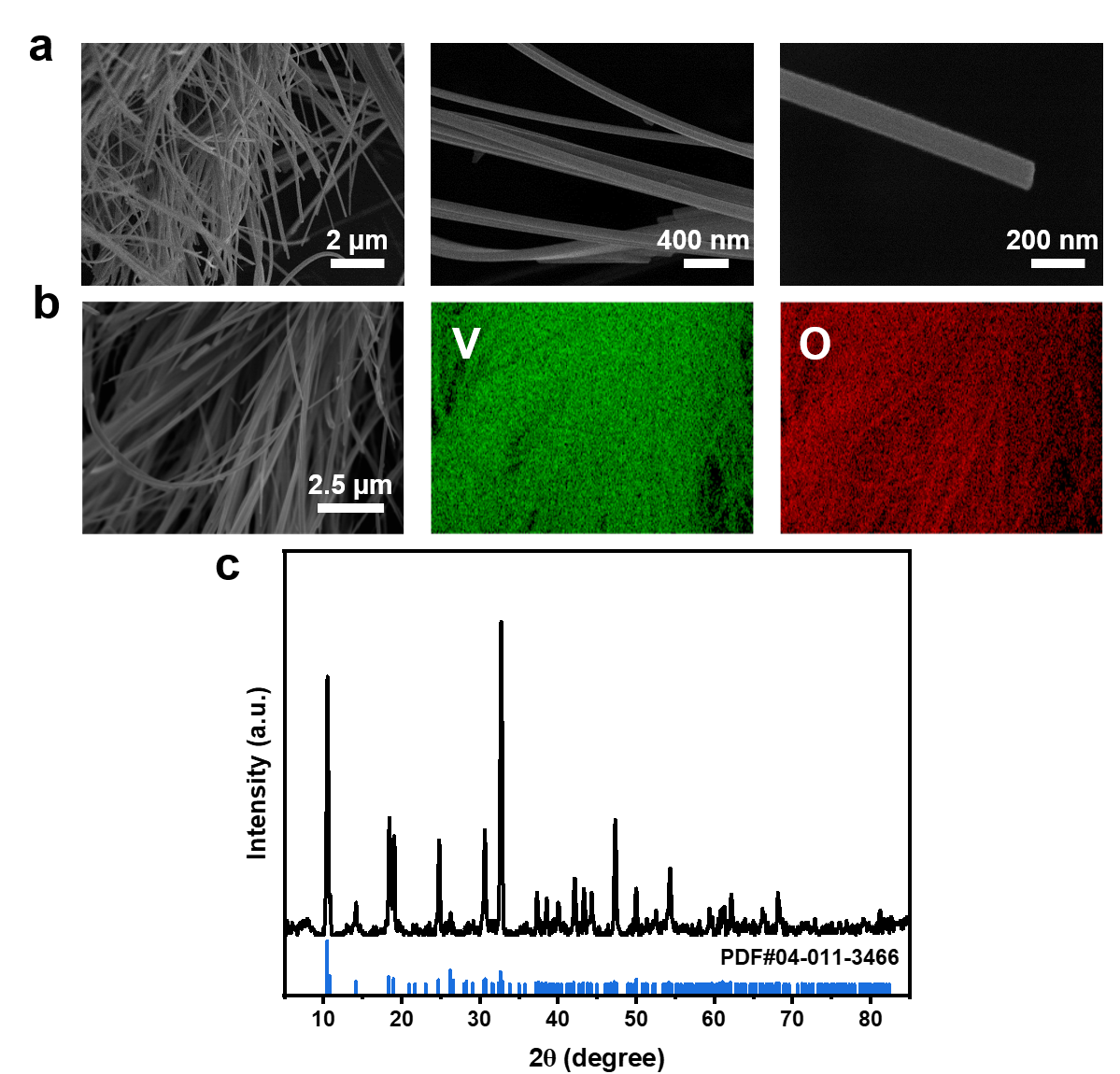


**Fig. S49** (**a**) SEM images, (**b**) EDS, and (**c**) XRD pattern of H_2_V_3_O_8_


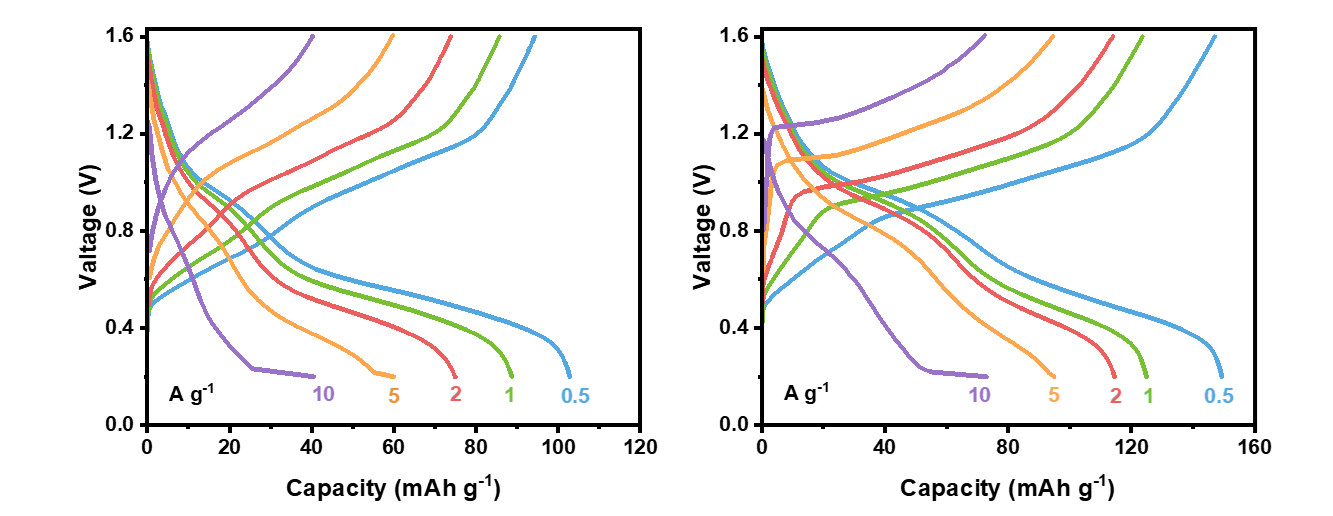


**Fig. S50** Galvanostatic charge–discharge curves of (**a**) Bare Zn||H_2_V_3_O_8_, (**b**) OIL@Zn||H_2_V_3_O_8_ coin cell at different rates


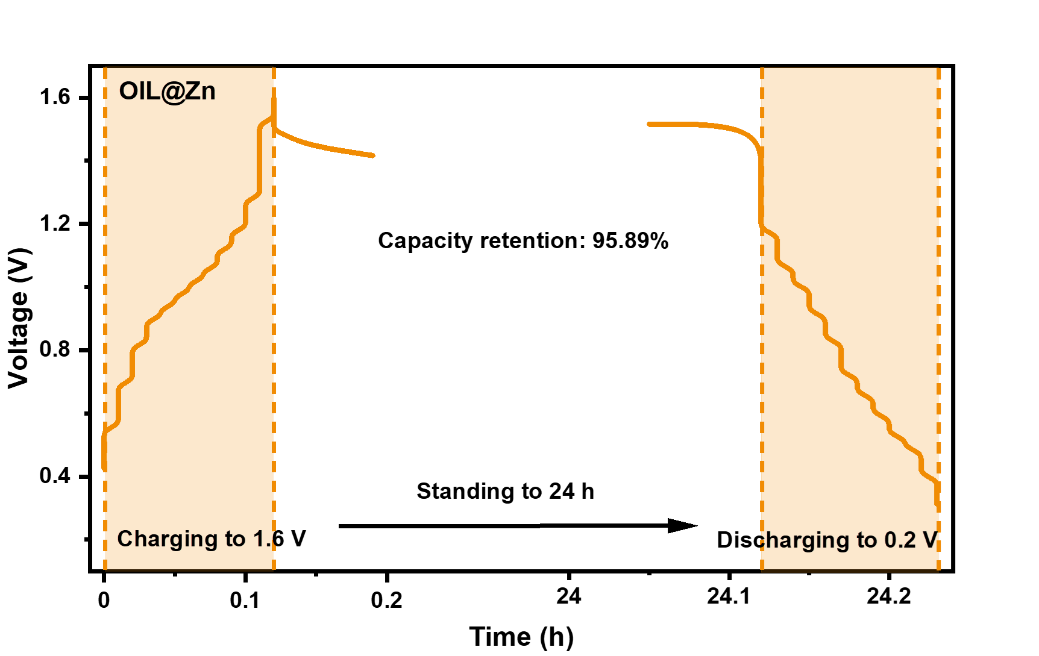


**Fig. S51** Self-discharge behavior of OIL@Zn||H_2_V_3_O_8_. The cells were subjected to charging at 5 A g^-1^ to 1.6 V, standing for 24 h, and then discharging to 0.2 V

**Table S1** EA test results of IPS

| C (%) | N (%) | H (%) | S (%) |
| --- | --- | --- | --- |
| 42.762 | 11.788 | 6.697 | 14.273 |

**Table S2** Corresponding wt% of EDS maps

| Sample | Element | Line type | wt% | wt% Sigma | Standard sample label |
| --- | --- | --- | --- | --- | --- |
|  | C | K | 34.84 | 1.27 | C Vit |
|  | N | K | 0.00 | 0.00 | BN |
| ADL@Zn | O | K | 56.48 | 1.64 | SiO_2_ |
|  | F | K | 8.68 | 2.19 | CaF_2_ |
|  | S | K | 0.00 | 0.00 | FeS_2_ |
| Bare Zn (IPS in electrolyte) | C  N  O  F  S | K  K  K  K  K | 34.07  0.00  52.93  12.37  0.00 | 1.54  0.00  1.93  2.66  0.00 | C Vit  BN  SiO_2_  CaF_2_  FeS_2_ |
|  | C | K | 26.43 | 0.57 | C Vit |
|  | N | K | 0.00 | 0.00 | BN |
| OIL@Zn | O | K | 64.18 | 0.84 | SiO_2_ |
|  | F | K | 8.66 | 1.00 | CaF_2_ |
|  | S | K | 0.72 | 0.17 | FeS_2_ |
|  |  |  |  |  |  |
|  | C | K | 25.4 | 3.1 | C Vit |
|  | N | K | 2.0 | 6.4 | BN |
| OIL-IPS@Zn | O | K | 43.4 | 4.1 | SiO_2_ |
|  | F | K | 28.8 | 4.2 | CaF_2_ |
| H_2_V_3_O_8_ | S  V  O | K  K  K | 0.4  57.63  42.37 | 0.4  0.58  0.58 | FeS_2_  V  SiO_2_ |

**Table S3** Atomic (%) of elements of ADL@Zn, OIL@Zn and OIL-IPS@Zn measured by XPS at varying depths

|  | ADL@Zn | | OIL@Zn | | OIL-IPS@Zn | |
| --- | --- | --- | --- | --- | --- | --- |
| Depth | 0 nm | 10 nm | 0 nm | 10 nm | 0 nm | 10 nm |
| Zn | 48.25 | 64.04 | 43.45 | 62.30 | 61.96 | 27.13 |
| C | 14.50 | 5.44 | 18.88 | 6.93 | 14.37 | 44.59 |
| O | 35.68 | 29.65 | 35.39 | 29.8 | 20.74 | 25.56 |
| N | --- | --- | --- | --- | 0.73 | 0.43 |
| F | 0.29 | 0.27 | 0.55 | 0.09 | 0.32 | 0.35 |
| S | 1.28 | 0.61 | 1.72 | 0.89 | 1.89 | 1.94 |

**Table S4** Atomic (%) of elements of OIL-IPS@Zn (after 200 h) measured by XPS at varying depths

| Depth | Zn | C | O | N | S |
| --- | --- | --- | --- | --- | --- |
| 0 nm | 48.12 | 17.26 | 33.39 | 1.24 | --- |
| 10 nm | 62.86 | 6.04 | 28.21 | 0.59 | 2.31 |

**Table S5** Compared with published performance

| Electrode | Electrolyte | Current  density  (mA cm^-2^) | Capacity  (mAh cm^-2^) | Lifetime  (h) | Refs. |
| --- | --- | --- | --- | --- | --- |
| OIL-IPS@Zn | 0.5 M Zn(OTf)_2_ | 1  50 | 1  10 | 3523  3284 | This work |
| MB@Zn | 2 M ZnSO_4_ | 0.2  0.5  2 | 0.2  0.25  1 | 1600  1600  650 | [S1] |
| PVDF-Sn@Zn | 2 M ZnSO_4_ | 1  5  10 | 1  5  10 | 1200  500  200 | [S2] |
| PD-Zn | 2 M ZnSO_4_ | 5  7.5  10 | 1  1  1 | 1000  700  500 | [S3] |
| TW20 | 2 M ZnSO_4_ | 1  5 | 1  1 | 2500  500 | [S4] |
| Xylitol | 2 M ZnSO_4_ | 1  5 | 1  1 | 1100  1000 | [S5] |
| HZF/Zn | 2 M ZnSO_4_ | 1  10 | 1  1 | 2600  1100 | [S6] |
| MPVMT | 2 M ZnSO_4_ | 1  5  50 | 1  5  1 | 2000  680  300 | [S7] |
| ZnSe@Zn | 2 M ZnSO_4_ | 1  10  30 | 1  10  10 | 1530  260  172 | [S8] |
| 3D-Zn | TBA | 5 | 5 | 160 | [S9] |
| AAn-COF | 1 M ZnSO_4_ | 5  10  20 | 1  1  1 | 400  300  300 | [S10] |
| NGO@Zn | 2 M ZnSO_4_ | 1  5 | 1  5 | 1200  300 | [S11] |
| Sn@Zn | 2 M ZnSO_4_ | 1 | 1 | 1500 | [S12] |
| D-HfO_2_-x@Zn | 2 M ZnSO_4_ | 5 | 1 | 2100 | [S13] |
| HG-002-Zn | 2 M ZnSO_4_ | 20  30 | 20  15 | 210  260 | [S14] |

**Table S6** Compared with published full cell performance

| Electrode | Current  density  (A g^-1^) | Capacity  (mAh g^-1^) | Cycle number | Refs. |
| --- | --- | --- | --- | --- |
| OIL-IPS@Zn\|\|H_2_V_3_O_8_ | 5  10 | 126  89.7 | 3130  7300 | This work |
| Zn@Bi/Bi_2_O_3_\|\|MnO_2_ | 2 | 133 | 1200 | [S15] |
| Zn-PA@Zn\|\|MnO_2_ | 2 | 150 | 600 | [S16] |
| PVA@Zn\|\|V_2_O_5_ | 3 | --- | 500 | [S17] |
| HZF/Zn\|\|MnO_2_ | 1 | 182 | 2000 | [S6] |
| Zn\|\|ZMOQD@C | 1 | 143.9 | 1500 | [S18] |
| 3D-Zn(002)\|\|NH_4_V_4_O_10_ | 5  2 | 96.7  114.5 | 4000  1500 | [S19] |
| Zn@Cu\|\|MnO_2_ | 0.5  2 | 192  141 | 300  100 | [S20] |
| Zn\|\|V_2_O_5_ | 1 | 113 | 1000 | [S21] |
| Zn\|\|MnO_2_ | 2 | 200 | 700 | [S22] |
| Zn\|\|PANI | 1 | 100 | 1000 | [S23] |
| Zn\|\|NaV_3_O_8_·1.5H_2_O | 0.5  10 | 330  337 | 550  1200 | [S24] |

**Supplementary References**

1. T. Huang, K. Xu, N. Jia, L. Yang, H. Liu et al., Intrinsic interfacial dynamic engineering of zincophilic microbrushes via regulating Zn deposition for highly reversible aqueous zinc ion battery. Adv. Mater.  **35**, 2205206 (2023). <https://doi.org/10.1002/adma.202205206>.
2. Q. Cao, Y. Gao, J. Pu, X. Zhao, Y. Wang et al., Gradient design of imprinted anode for stable Zn-ion batteries. Nat. Commun.  **14**, 641 (2023). <https://doi.org/10.1038/s41467-023-36386-3>.
3. Q. Li, A. Chen, D. Wang, Y. Zhao, X. Wang et al., Tailoring the metal electrode morphology via electrochemical protocol optimization for long-lasting aqueous zinc batteries. Nat. Commun. **13**, 3699 (2022). <https://doi.org/10.1038/s41467-022-31461-7>.
4. Q. Deng, S. You, W. Min, Y. Xu, W. Lin et al., Polymer molecules adsorption-induced zincophilic-hydrophobic protective layer enables highly stable Zn metal anodes. Adv. Mater. **36**, 2312924 (2024). <https://doi.org/10.1002/adma.202312924>.
5. H. Wang, W. Ye, B. Yin, K. Wang, M. S. Riaz et al., Modulating cation migration and deposition with xylitol additive and oriented reconstruction of hydrogen bonds for stable zinc anodes. Angew. Chem. Int. Ed. **62**, e202218872 (2023). <https://doi.org/10.1002/anie.202218872>.
6. F. Duan, X. Yin, J. Ba, J. Li, Y. Yu et al., A hydrophobic and zincophilic interfacial nanofilm as a protective layer for stable Zn anodes. Adv. Funct. Mater. **34**, 2310342 (2024). <https://doi.org/10.1002/adfm.202310342>.
7. Z. Zheng, X. Zhong, Q. Zhang, M. Zhang, L. Dai et al., An extended substrate screening strategy enabling a low lattice mismatch for highly reversible zinc anodes. Nat. Commun. **15**, 753 (2024). <https://doi.org/10.1038/s41467-024-44893-0>.
8. X. Yang, C. Li, Z. Sun, S. Yang, Z. Shi et al., Interfacial manipulation via in situ grown ZnSe cultivator toward highly reversible Zn metal anodes. Adv. Mater. **33**, 2105951 (2021). <https://doi.org/10.1002/adma.202105951>.
9. A. Bayaguud, X. Luo, Y. Fu, C. Zhu, Cationic surfactant-type electrolyte additive enables three-dimensional dendrite-free zinc anode for stable zinc-ion batteries. ACS Energy Lett.  **5**, 3012–3020 (2020). <https://doi.org/10.1021/acsenergylett.0c01792>.
10. C. Guo, J. Zhou, Y. Chen, H. Zhuang, Q. Li et al., Synergistic manipulation of hydrogen evolution and zinc ion flux in metal-covalent organic frameworks for dendrite-free Zn-based aqueous batteries. Angew. Chem. Int. Ed. **61**, e202210871 (2022). <https://doi.org/10.1002/anie.202210871>.
11. J. Zhou, M. Xie, F. Wu, Y. Mei, Y. Hao et al., Ultrathin surface coating of nitrogen-doped graphene enables stable zinc anodes for aqueous zinc-ion batteries. Adv. Mater. **33**, 2101649 (2021). <https://doi.org/10.1002/adma.202101649>.
12. J. Li, Z. Liu, S. Han, P. Zhou, B. Lu et al., Hetero nucleus growth stabilizing zinc anode for high-biosecurity zinc-ion batteries. Nano-Micro Lett. **15**, 237 (2023). <https://doi.org/10.1007/s40820-023-01206-2>.
13. K. Zhang, C. Li, J. Liu, S. Zhang, M. Wang et al., Defect-rich functional HfO2-x for highly reversible Zn metal anode. Small **20**, 2306406 (2024). <https://doi.org/10.1002/smll.202306406>.
14. Z. Chen, Q. Wu, X. Han, C. Wang, J. Chen et al., Converting commercial Zn foils into single (002)-textured Zn with millimeter-sized grains for highly reversible aqueous zinc batteries. Angew. Chem. Int. Ed. **63**, e202401507 (2024). <https://doi.org/10.1002/anie.202401507>.
15. X. Tian, Q. Zhao, M. Zhou, X. Huang, Y. Sun et al., Synergy of dendrites-impeded atomic clusters dissociation and side reactions suppressed inert interface protection for ultrastable Zn anode. Adv. Mater. **36**, 2400237 (2024). <https://doi.org/10.1002/adma.202400237>.
16. H. Liu, J.-G. Wang, W. Hua, L. Ren, H. Sun et al., Navigating fast and uniform zinc deposition via a versatile metal–organic complex interphase. Energy Environ. Sci. **15**, 1872–1881 (2022). <https://doi.org/10.1039/D2EE00209D>.
17. X. Chen, W. Li, S. Hu, N. G. Akhmedov, D. Reed et al., Polyvinyl alcohol coating induced preferred crystallographic orientation in aqueous zinc battery anodes. Nano Energy **98**, 107269 (2022). <https://doi.org/10.1016/j.nanoen.2022.107269>.
18. S. Deng, Z. Tie, F. Yue, H. Cao, M. Yao et al., Rational design of ZnMn_2_O_4_ quantum dots in a carbon framework for durable aqueous zinc-ion batteries. Angew. Chem. Int. Ed. **61**, e202115877 (2022). <https://doi.org/10.1002/anie.202115877>.
19. X. Chen, Z. Zhai, T. Yu, X. Liang, R. Huang et al., Constructing a 3D zinc anode exposing the Zn(002) plane for ultralong life zinc-ion batteries. Small **20**, 2401386 (2024). <https://doi.org/10.1002/smll.202401386>.
20. K. Yang, Z. Zhu, X. He, R. Song, X. Liao et al., High-performance zinc metal anode enabled by large-scale integration of superior ion transport layer. Chem. Eng. J. **492**, 152114 (2024). <https://doi.org/10.1016/j.cej.2024.152114>.
21. C. Yang, P. Woottapanit, S. Geng, K. Lolupiman, X. Zhang et al., Highly reversible Zn anode design through oriented ZnO(002) facets. Adv. Mater. **36**, 2408908 (2024). <https://doi.org/10.1002/adma.202408908>.
22. A. Herter, A. Shams-Ansari, F. F. Settembrini, H. K. Warner, J. Faist et al., Terahertz waveform synthesis in integrated thin-film lithium niobate platform. Nat. Commun. **14**, 11 (2023). <https://doi.org/10.1038/s41467-022-35517-6>.
23. X. Zhao, N. Dong, M. Yan, H. Pan, Unraveling the interphasial chemistry for highly reversible aqueous Zn ion batteries. ACS Appl. Mater. Interfaces **15**, 4053–4060 (2023). <https://doi.org/10.1021/acsami.2c19022>.
24. Y. Lv, M. Zhao, Y. Du, Y. Kang, Y. Xiao et al., Engineering a self-adaptive electric double layer on both electrodes for high-performance zinc metal batteries. Energy Environ. Sci. **15**, 4748–4760 (2022). <https://doi.org/10.1039/D2EE02687B>.
